# Lessons learned from using respondent-driven sampling (RDS) to assess sexual risk behaviors among Kenyan young adults living in urban slum settlements: A process evaluation

**DOI:** 10.1371/journal.pone.0231248

**Published:** 2020-04-10

**Authors:** Larissa Jennings Mayo-Wilson, Muthoni Mathai, Grace Yi, Margaret O. Mak’anyengo, Melissa Davoust, Massah L. Massaquoi, Stefan Baral, Fred M. Ssewamala, Nancy E. Glass, NAHEDO Study Group

**Affiliations:** 1 Department of Applied Health Sciences, Indiana University School of Public Health, Bloomington, Indiana, United States of America; 2 Department of International Health, Johns Hopkins University, Bloomberg School of Public Health, Baltimore, Maryland, United States of America; 3 Department of Psychiatry, University of Nairobi, College of Health Sciences, Kenyatta National Hospital, Nairobi, Kenya; 4 Department of Mental Health, National Health and Development Organization (NAHEDO), Kenyatta National Hospital, Nairobi, Kenya; 5 Department of Epidemiology, Johns Hopkins Bloomberg School of Public Health, Baltimore, Maryland, United States of America; 6 Washington University in St. Louis, The Brown School, Goldfarb, One Brookings, Drive, St. Louis, Missouri, United States of America; 7 Johns Hopkins University School of Nursing, Baltimore, Maryland, United States of America; University of Arkansas for Medical Sciences, UNITED STATES

## Abstract

**Background:**

Respondent-driven sampling (RDS) is a peer-referral sampling methodology used to estimate characteristics of underserved groups that cannot be randomly sampled. RDS has been implemented in several settings to identify hidden populations at risk for HIV, but few studies have reported the methodological lessons learned on RDS design and implementation for assessing sexual risk behaviors in marginalized youth.

**Methods:**

We used RDS to recruit N = 350 young adults, aged 18 to 22, who were living in urban slum settlements in Nairobi, Kenya. A structured survey was used to assess sexual risk behaviors. Twenty seeds were selected and asked to recruit up to three eligible peers. We used small monetary incentives and a three-day recruitment coupon with sequential numbers linking recruiters to their recruits.

**Results:**

Data collection was completed in 8 days with a maximum chain length of 6 waves. Each seed yielded 16 to 21 eligible recruits. Three (15%) seeds were unproductive and were replaced. RDS benefits were high identification rates (90% coupons returned per coupons given), high eligibility rates (100% eligible recruits per coupons returned), and high efficiency (~39 eligible recruits per day). 44% of the sample was female. Most recruits (74%) reported being “friends” for 7+ years with their recruiter. RDS overcame feasibility concerns of household-, clinic-, and school-based sampling methodologies in that underserved youth who were unemployed (68%), out of school (48%), ethnic minorities (26%), and having prior residential instability (≥2 moves in the past year) (20%) were successfully recruited, based on weighted analyses. Youth reporting HIV risk behaviors, including unprotected sex (38%), sex while high/drunk (35%), and sex exchange for pay (14%), were also enrolled. However, 28% were not sexually active within the last 6 months. Challenges included managing wait times during peaks and participant referral expectations. Community engagement, use of study-stamped coupons, broad inclusion criteria, incentives, and study sites within walking distances all contributed to the successful implementation of the sampling methodology.

**Conclusion:**

RDS is an important tool in reaching a diverse sample of underserved and at-risk young adults for study participation. Implications for optimizing RDS for behavioral studies in this population are discussed.

## Background

Approximately 70% of people living with HIV live in Sub-Saharan African countries [1], and young adults living in Africa’s urban slum settlements are disproportionately at risk for HIV infection [2]. Characterized by densely populated impoverished communities, urban slum settlements in many African countries lack basic services and infrastructure such as electricity, durable housing, and clean water [3, 4]. The majority of urban slum residents rely on low-skilled work (i.e., domestic labor, casual jobs, petty trading) in response to high levels of unemployment and income instability [3, 4]. In Kenya, over half of Kenya’s city dwellers live in slums [3]. Recent data estimates that the HIV prevalence in Kenya’s urban slum settlements is 12% compared to 5% in non-slum urban areas and is transmitted primarily through heterosexual contact [2, 4]. In addition, Kenyan young adults within the lowest wealth quintile and in urban areas have higher HIV prevalence (3.1% and 7.2%) in comparison to wealthier young adults (2.6%) living in rural areas (6.0%) [5]. Young adults living in urban slum settlements engage frequently in sexual risk behaviors, such as condomless sex, transactional sex, or sex with multiple concurrent sexual partners that result in higher risk of HIV and other sexually transmitted infections [6–9]. In addition to being at increased risk for HIV, young adults living in urban slum settlements are often hidden and not well reached using traditional surveillance systems [10]. School-based sampling strategies in urban slum settings with absenteeism and high drop-out rates result in biased estimates that exclude out-of-school youth, including newly-emerging young adults [11, 12]. Much of our knowledge regarding sexual behaviors in young adults is generated from household-based sampling strategies, such as the Demographic and Health Survey (DHS) [13]. However, young adults in urban slum settings risk being inaccessible via households due to housing instability, migration, conflict, or spending significant time away from home in search of employment, which may hinder their ability to connect with other social groups [12]. In addition, young women in Kenya’s urban slum settings may not reveal all of their sexual behaviors during HIV surveillance activities at antenatal clinics, which exclude young men [10]. The lack of unbiased global data that adequately includes young adults in urban slum settings who are out-of-school, away from home, and disconnected from health facilities has led to increased interest in respondent-driven sampling (RDS) to reach a larger number of impoverished and underserved youth who are often missed using more traditional sampling methodologies [14].

RDS is a sampling method that operates as a chain-referral sampling process that is driven by peers and used to estimate characteristics of hidden groups that public health services do not well reach [15, 16]. It is similar to snowball sampling used primarily in qualitative research in that it begins with a convenience sample of purposively selected seeds [17, 18]. However, unlike snowball sampling, RDS produces more reliable data estimates by using a “link-tracing design” that estimates participant network sizes and calculates selection probabilities between recruiters and their recruits [17, 18]. For each consecutive wave, RDS also yields a larger and more externally valid study sample that is not restricted to frequenting a particular study site [17–19]. RDS is recommended for individuals who are moderately networked, but cannot be randomly sampled [17, 18]. This is applicable to urban slum young adults in that they are connected through a network of friends and sexual partners, and can more readily identify community peers engaging in similar sexual risk behaviors. However, generating an exhaustive sampling frame (as required by traditional statistical methods) would be infeasible in many young adult slum settings due to the high mobility and disconnectedness of urban slum youth, and the potentially stigmatizing consequences of enumerating young adults living in impoverished communities [17, 18, 20]. Such young adults may also be more receptive to requests for study participation from peers as compared to older and/or outside researchers [17, 18]. Finally, efforts to understand and address HIV outcomes in urban slum settlements are hindered by a dearth of data necessary to generate urban slum-specific estimates [17]. RDS may therefore provide an important means for contributing to this area of research. However, like all sampling strategies, RDS relies on a specific set of assumptions that may or may not be implementable in urban slum settings. The assumptions are that (i) respondents know each other by name and are members of a defined network; (ii) respondents can accurately report their personal network size, defined as the number of individuals they know who know them within the target group; (iii) respondents’ sampling occurs with replacement; and (iv) respondents select recruits randomly within their networks [18, 21, 22].

The aim of this paper is to describe the implementation experience and lessons learned from using RDS to assess sexual risk behaviors in Kenyan young adults living in urban slum settlements, including the extent to which RDS assumptions were achieved by urban slum young adults. RDS has been widely used in HIV biological and behavioral studies [23, 24] to recruit key and hidden populations such as men who have sex with men (MSM) [10, 19, 25–29], people who inject drugs [10, 19, 30], and female sex workers (FSW) [10, 19, 31–34]. The method has also recently been implemented to recruit other underserved groups at risk for HIV, including migrant workers [35] and youth experiencing homelessness [12, 36–38]. However, to our knowledge, no studies have examined the feasibility of using RDS in a sub-Saharan African setting to recruit young adults at risk for HIV within urban slum settlements. As a result, less is known regarding the acceptability, safety, and efficacy of RDS for this population. Additionally, while there is an abundance of literature reporting results from studies using RDS, there are fewer papers describing implementation successes and challenges in this population [10, 12, 39]. This paper discusses positive and negative aspects of the RDS process, including operational changes made to address challenges and enhance overall efficacy in this population.

## Methods

### Design

A cross-sectional survey was administered using RDS within two urban slum settlements in Nairobi, Kenya: Korogocho and Kawangwere. The survey was administered in-person in English or the local language (Kiswahili) by nine trained Kenyan interviewers to young adults, aged 18 to 22, who were living in one of the two settlements at the time of the study.

### Setting

Korogocho is one Nairobi’s northeast urban slums, and Kawangware, the city’s western urban slum. Both are located within 6 to 9 miles from the city center. According to the most recent Population and Housing Census, each slum’s population is approximately 130,000 to 200,000 people, the majority of whom are children and young adults. There is little infrastructure serving Korogocho and Kawangware’s residents, including limited safe drinking water, sanitation, and lighting [40].

### Eligibility

Study eligibility was determined using a paper-based screening tool. Individuals were eligible if, at the time of enrollment, they self-reported that they: were Kenyan, had primary residence within the Korogocho or Kawangware urban slum settlements, and were aged 18–22 years. Self-report was used given that young adults in this population commonly lack government-issued identification or postal mail. Therefore, to minimize sample biases, in addition to self-report, a community health worker from the urban slum settlement was present during enrollment to affirm residence and age. In addition, participant names and birth dates were used to avoid duplication. All three eligibility criteria had to be met to be included in the study. The screening process took approximately two minutes to complete and was conducted by the field supervisor who then assigned the participant to an on-site interviewer.

### Sample size

A target sample of 350 young adults was determined to allow for adequate power and precision in estimating an expected average prevalence of condomless sex during last sexual intercourse of 41%. The expected prevalence was based on the 2014 Demographic and Health Survey (DHS) data from Kenya which found that 56.7% and 73.9% of Kenyan women, aged 20–24 and 15–19, respectively and 29.8% and 35.9% of Kenyan men, aged 20–24 and 15–19, respectively, reported not using a condom during last sexual intercourse [13, 41]. The prevalence estimates of the RDS approach are discussed in a forthcoming paper.

### Seed selection

To initiate the RDS process, 20 seeds (initial recruiters) were purposefully selected from central areas of the two urban slum settlements. The study team relied on the local knowledge of two young adult representatives from each settlement (representing a total of 4) to identify initial seeds. These representatives were selected by a community health worker who had prior experience in both urban slum settlements. Each representative was selected based on his/her in-depth knowledge of the households within the urban slum settlement. The representatives were informed of the goals of the study and the study’s eligibility criteria. Each representative was paid 2,000 Kenyan shillings (equivalent to $20.00 USD). They were not eligible to participate in the study. The representatives then explained to the initial seeds the study’s purpose and referred them to the interviewers on the same or following day. The initial seed participants (wave 0) were screened for eligibility, administered informed consent, and provided the survey. They were then asked to recruit three Kenyan peers, aged 18 to 22, who they know who currently lived within their urban slum settlement (Korogocho or Kawangware). All 20 seeds were invited to begin recruiting in the same week.

### RDS recruits

The initial seeds (wave 0) and subsequent peer recruits (waves 1 to 6) were each given recruitment coupon(s) to invite additional young adults into the study. Recruited participants were asked to meet the trained interviewers at the community center within their urban slum settlement on the afternoon of their recruitment or in the morning of the next day. The recruited participants were then screened for eligibility, administered informed consent, provided the survey, and invited to recruit additional Kenyan peers, aged 18 to 22, who they know who currently lived within their urban slum settlement. Recruiting participants were advised that if more than one individual within a household fit the preferred characteristic(s), s/he should provide a recruitment coupon to a maximum of one peer.

### Coupon and payment

**Fig 1:**
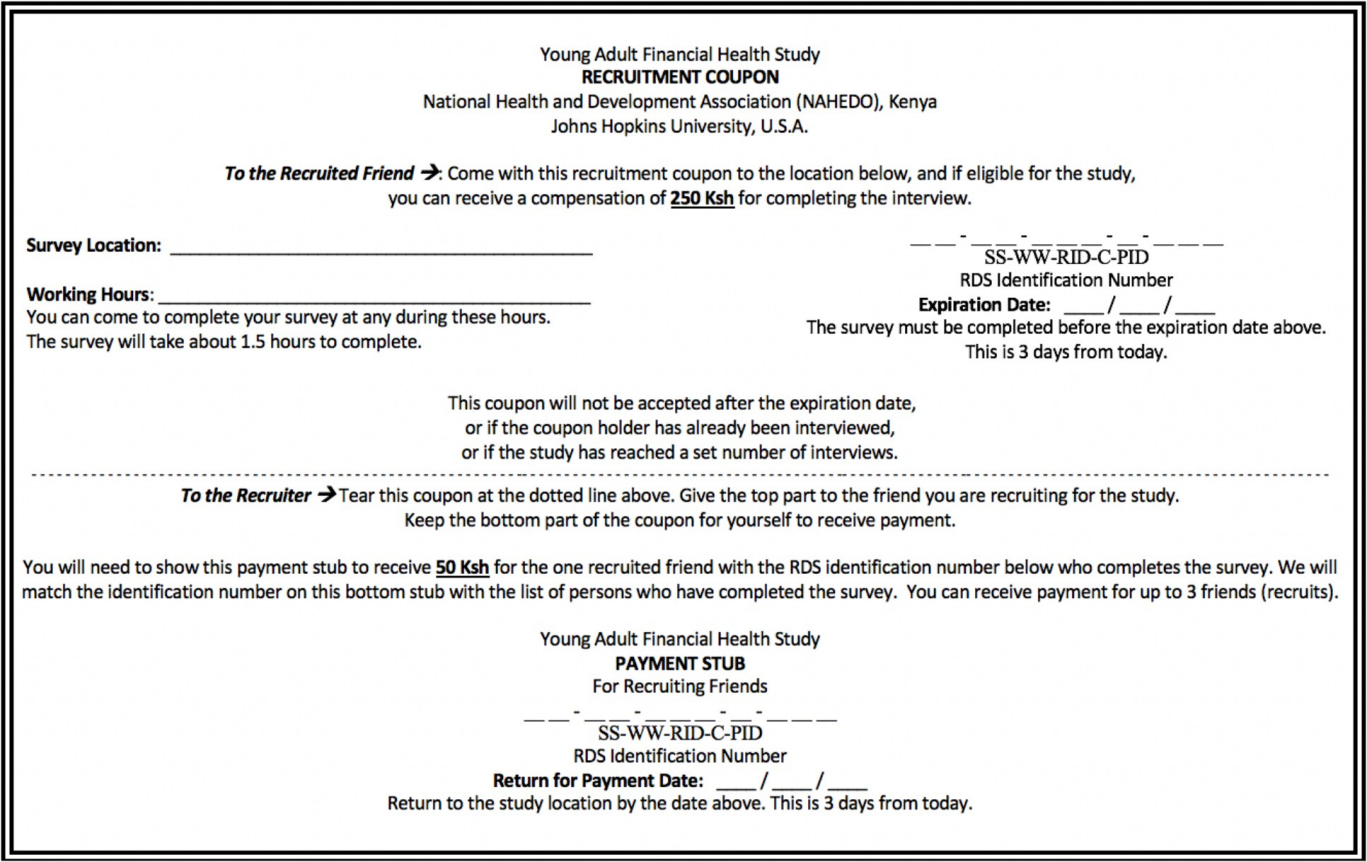
Recruitment coupon and payment stub provided to participants to identify eligible peers (English version).

Fig 1 shows the English version of the RDS recruitment coupon that was used in the study. The coupon was also printed in Kiswahili. The recruitment coupon included an expiration date (i.e., three days later), after which it could not be used. This was done in order to manage the study’s recruitment pace and data collection end date. The coupon also contained sequential numbers in order to link the participants to prior recruits’ study identification number (ID) in order to understand the recruitment chain. Each participant was paid 250 Kenyan Shillings (the approximate equivalent to $2.50 USD) for completing the study survey. They were also paid an additional 50 Kenyan Shillings (the approximate equivalent to $0.50 USD) for each individual they recruited to the study, up to three individuals. Payment for study participation was provided in cash at the end of the survey. Payment for recruitment referral was tabulated by submitted coupon of the recruit and provided in cash at the time of recruit’s completed survey or any subsequent day during the data collection period. The trigger for recruitment-based compensation was that the recruit completed the survey. Recruiters were asked to return to the study site approximately three days later (on or about the coupon’s expiration date) to be paid. We then matched the returned coupon stub(s) from the recruit(s) to the coupon number of the recruiter. The total possible payment for participants completing was 450 Kenyan Shillings (the approximate equivalent to $4.50 USD). All participants were oriented to the coupon to facilitate understanding and were informed that recruiting was optional. Decisions not to recruit did not affect a participant’s payment for completing the survey.

### Survey data collection

Data were collected in April 2017. Interviews were conducted by one of nine interviewers who had prior experience in conducting survey research in Nairobi and who underwent a three-day training process led by the study’s co-principal investigators (LJMW and MM). A large number of interviewers were used to facilitate a rapid pace of data collection. Interviewers remained on-site throughout the day to be readily available for presenting recruits. Interviewers were also in close contact with two field site managers and two study authors (LJMW, MM) who visited both sites each day and/or communicated by cell phone throughout the day to the site managers. All interviewers used a structured questionnaire, which was developed by the research team and pre-tested using the 17 young adults (not included in the study). The survey questionnaire was developed using sexual behavioral and economic variables previously used in prior studies with youth, lower-income, vulnerable groups, and/or sub-Saharan African populations. It included questions relating to sexual risk behaviors relating to the primary outcome of survey, as well as HIV prevention practices, HIV-related care-seeking, demographic status, financial history, savings, and other economic measures. The survey was translated into Kiswahili. All interviews were conducted privately in English or Kiswahili, and lasted about 40 minutes.

### RDS process measures

The primary outcome of this study included RDS process measures. The measures used to describe the RDS recruitment process were: network size of participants’ peers, recruiter-recruit relationship, participant safety, identification rate, recruitment efficiency (including maximum chain length and screening threshold), and sample equilibrium.

To measure *network size of participants* as relating to their selection probability, we asked, “How many young adults, aged 18 and older, are there that you know and that know you, and that live in this urban settlement?” Following this question, we asked, “How many of them are aged 18 to 22?”. To measure the *nature of the relationship between the recruiter and his/her recruit*, each participant was asked, “What is your relationship to the person who gave you the study’s recruitment coupon?” The response options were: friend, family member, neighbor, no relationship, or other. Participants were also asked, “how many years have you known this person?” and “where did you first meet the person who recruited you?”. The response options were in community, at school, at work or job, at religious gathering, or other. To determine whether participants were pressured to participate by their recruiter, we also asked, “How many times did this person (e.g., your recruiter) remind you to participate in the study?” We also asked, “How would you describe the nature of the invitation from your recruit?” Responses options were to select all that apply and included: friendly, aggressive/pushy, exciting, worrisome, or other(specify).

Three questions were used at the end of each survey to measure *participant safety*. To identify any prior experience or anticipation of violence, we asked, “Did you experience any past threats to your safety as a result of participating in this study?” (Yes/No) and “Do you expect that you will experience any future threats to your safety as a result of participating in this study?” (Yes/No). As a final question, to assess whether participants had any concerns related to recruiting, we asked, “How willing are you to distribute coupons for this study to your peers?” in which responses were given using a scale of 0 to 10 where 0 = "not willing" and 10 = "very willing". To measure *identification rates*, we calculated the number of coupons distributed and the number of coupons returned to determine the overall and by site number of coupons returned per coupons distributed (i.e., coupon return rate). Coupon distribution and returns were documented at the time of enrollment by one of two site managers using a paper-based tracking log.

For *recruitment efficiency*, we measured the number of days receiving recruits, the initial and cumulative number of eligible recruits per days elapsed, the mean number of recruits per day by site, gender and total sample, and the mean maximum number of days from the recruiter’s survey to his/her recruit’s survey. We also tracked the number of recruits enrolled by a single seed, the screening threshold (defined as the number of screened recruits per enrolled participants), and the maximum chain length needed (defined as the number of waves required by site and total sample to achieve the target sample size). Gender was used to assess *sample equilibrium*, which we measured by calculating the cumulative sample proportion at each wave for each site. When the proportion was within 2% of the final sample proportion at a particular wave (without fluctuation), that wave was labeled as the equilibrium point [15, 42].

### Analysis

The analysis was conducted in three stages. First, we created a database in Excel with participant RDS tracking information. This included recruitment study site, wave number, the participant’s enrollment date, his/her study ID, the study ID of his/her recruiter, the number of coupons distributed, the number of coupons returned (from his/her recruits), and whether the recruiter was paid for successful recruits. We then linked all recruiters to their recruits and calculated the study’s identification and efficiency rates, including RDS measures relating to recruiter relationship and safety. Excel was also used to create line graphs of the daily recruitment and cumulative equilibrium. Second, we analyzed participants’ survey responses using STATA SE (Version 15) (Statacorp, LP, College Station, TX). We calculated the crude and RDS-adjusted prevalence estimates and 95% confidence intervals (CIs) for each of the demographic characteristics and sexual behavior responses. The RDS-adjusted weights were generated using the RDS-II estimator in the Respondent Driven Sampling Analysis Tool (RDSAT), Version 7.146 (Cornell University, Ithaca, NY, U.S., http://www.respondentdrivensampling.org), based on the inverse of each participant’s personal network size, rescaled to sum to the total sample size [43]. Where applicable, we used the median reported network size for participants who responded “very many” or “uncountable” regarding the number of similarly-aged peers that they knew in the urban slum settlement. All analyses were considered statistically significant at p<0.05. Missing data were excluded from the reported statistics. As a third and final step, we created a graphical presentation of the recruitment networks generated at each site using NetDraw (Version 2.160, Lexington, KY, U.S., https://sites.google.com/site/netdrawsoftware/home) [44].

### Ethical considerations

This study received ethics approval from the Johns Hopkins Bloomberg School of Public Health Institutional Review Board (IRB#0007421) and the Kenyatta National Hospital, University of Nairobi Ethics and Research Review Committee.

## Results

### RDS process measures

Table 1 describes the outcomes of the RDS process relating to sample size, number of seeds, network size, recruiter-recruit relationship, identification, efficiency, and safety. A total of N = 369 young adults were enrolled in the study (Table 1). Nineteen (n = 19) participants were excluded in sum due to: being a part of the survey pre-test prior to initiating RDS (n = 17) or having a duplicate entry (n = 2). This resulted in a final analytical sample of N = 350, representing 100% of the target sample size. Twenty (n = 20) seeds were selected at the start of the study (i.e., 10 seeds per site with equal numbers of young men and women). At one site, 3 (30%) of the original 10 seeds were unproductive. Two of the unproductive seeds were female and were replaced by male seeds, and one unproductive seed was male and was also replaced by a male seed. The 3 male replacement seeds successfully obtained recruits. A total of 20 productive seeds (17 original and 3 replacement) were used in the study, accounting for 12 male seeds and 8 female seeds. Each seed yielded 16 to 21 recruits with a maximum chain length of 6 (Table 1).

**Table 1:** Respondent-driven sampling process measures by total sample, study site, and gender.

N (%)^a^	Total	Korogocho			Kawangwere		
		M	F	Sub-Total	M	F	Sub-Total
**Sample size**							
Total # participants enrolled in study	369	95	89	184	115	70	185
# survey pre-testers (prior to RDS start)	17	5	2	7	8	2	10
# duplicates	2	1	1	2	0	0	0
Total # participants in final analytical sample	350	89	86	175	107	68	175
**Seeds**							
# original seeds (at start of study)	20	5	5	10	5	5	10
# original productive seeds	17	5	5	10	4	3	7
# original unproductive seeds	3	0	0	0	1	2	3
# replacement seeds	3	0	0	0	3	0	3
# productive replacement seeds	3	0	0	0	3	0	3
# unproductive replacement seeds	0	0	0	0	0	0	0
Total # productive seeds (original+replacement)	20	5	5	10	7	3	10
# recruits enrolled by a single seed (range)	16–21	17–21	16–19	16–21	17–21	17–20	17–21
# recruitment waves (excluding seeds)	6	4	4	4	6	6	6
**Recruit network size**							
Mean # all adults (aged ≥18 years) who participants knew in settlement (± SD)	25.7 (±34.4)	31.5 (±35.2)	27.3 (±31.6)	29.5 (±33.4)	26.9 (±38.4)	14.1 (±27.4)	21.9 (±35.0)
Mean # young adults (aged 18–22) who participants knew in settlement (± SD)	19.0 (±30.2)	17.8 (±24.2)	15 (±25.6)	16.4 (±24.9)	26.3 (±37.9)	14.1 (±27.4)	21.6 (±34.6)
# (%) reporting peer network size (aged 18–22)							
1–25 peers	262 (75%)	60 (17%)	67 (19%)	127 (36%)	76 (22%)	59 (17%)	135 (39%)
26–50 peers	30 (9%)	8 (2%)	5 (1%)	13 (4%)	14 (4%)	3 (1%)	17 (5%)
51–100 peers	19 (5%)	6 (2%)	3 (1%)	9 (3%)	9 (3%)	1 (0%)	10 (3%)
101–200 peers	8 (2%)	1 (0%)	1 (0%)	2 (1%)	4 (1%)	2 (1%)	6 (2%)
Unable to quantify (median used)	31 (9%)	14 (4%)	10 (3%)	24 (7%)	4 (1%)	3 (1%)	7 (2%)
**Recruiter-recruit relationship**							
Type of relationship							
Friend	258 (74%)	61 (68%)	60 (79%)	121 (69%)	90 (84%)	47 (69%)	137 (78%)
Relative	32 (9%)	11 (12%)	5 (6%)	16 (9%)	5 (5%)	11 (16%)	16 (9%)
Neighbor	49 (14%)	13 (15%)	16 (19%)	29 (17%)	11 (10%)	9 (13%)	20 (11%)
No relationship	9 (3%)	3 (3%)	4 (5%)	7 (4%)	1 (1%)	1 (1%)	2 (1%)
Mean # years knowing the recruiter (±SD)	7.3 (±7.4)	8.9 (±6.6)	6.7 (±6.5)	7.8 (±6.6)	6.6 (±5.7)	6.2 (±6.3)	6.5 (±5.9)
# (%) of recruits with same gender as recruiter	212 (61%)	62 (70%)	49 (56%)	111 (63%)	63 (59%)	38 (56%)	101 (58%)
Where first met recruiter							
Settlement	83%	93%	94%	94%	74%	74%	73%
School	6%	2%	0	1%	13%	7%	11%
Job	1%	0	2%	1%	0	0	2%
Religious gathering	1%	2%	0	1%	2%	1%	13%
Other	9%	2%	1%	2%	11%	18%	1%
Mean # times reminded to participate by recruiter (±SD)	1.9 (±1.2)	1.9 (±1.2)	2.0 (±1.6)	2.0 (±1.4)	1.9 (±1.1)	1.9 (±0.9)	1.9 (±1.0)
Nature of recruiter’s invitation							
Friendly	78%	74%	79%	77%	78%	80%	79%
Aggressive	0	1%	0	1%	0	0	0
Exciting	21%	22%	20%	21%	21%	19%	20%
Worrisome	0	1%	0	1%	0	0	0
Other	0	1%	0	1%	0	0	0
**Identification**							
# of participants eligible to receive coupons	197	52	46	98	55	44	99
% of eligible who agreed to distribute coupons^b^	96%	98%	98%	98%	91%	98%	94%
# participants receiving each # coupons							
3 coupons	80	24	16	40	19	21	40
2 coupons	0	0	0	0	0	0	0
1 coupon	117	28	30	58	36	23	59
0 coupons	153	37	40	77	52	24	76
Total # coupons distributed^d^	365	100	78	178	96	91	187
Total # coupons returned^e^	327	94	71	165	84	78	162
% coupons returned per distributed	90%	94%	91%	93%	88%	86%	87%
% eligible recruits among return coupons	100%	100%	100%	100%	100%	100%	100%
**Efficiency**							
# days receiving recruits	8	8	8	8	8	8	8
Mean # recruits per day (±SD)	39.0 (±16.7)	10.0 (±5.5)	9.7 (±7.3)	19.7 (±10.0)	13.3 (±6.8)	8.5 (±5.7)	21.8 (±8.6)
Cumulative # eligible recruits per days elapsed							
1 day	37	14	6	20	9	8	17
2 days	91	30	16	46	25	20	45
3 days	147	37	35	72	54	21	75
4 days	175	53	43	96	57	22	79
5 days	215	69	49	118	70	27	97
6 days	270	81	69	150	80	40	120
7 days	321	87	84	171	94	56	150
8 days	350	89	86	175	107	68	175
# participants in final analytical sample^c^	350	89	86	175	107	68	175
% analytical sample^c^	100%	25%	25%	50%	31%	19%	50%
Mean maximum # (± SD) weekdays for all of a recruiter’s recruit(s) to a complete survey^b^	3.0 (±2.4)	3.3 (±2.7)	3.0 (±2.0)	3.2 (±2.4)	2.7 (±2.1)	3.0 (±2.5)	2.8 (±2.3)
**Participant safety**							
# (%) reporting prior or future safety concerns as a result of study participation	2 (0.6%)	1 (0.6%)	1 (0.6%)	2 (1.1%)	0	0	0
Mean rated willingness to distribute coupons^b^–[Scale 0 to 10 = very willing]	8.7	9.0	8.6	8.8	8.8	8.5	8.6

^[a]^ Percentages not adding to 100 are due to missing or don’t know responses.

^[b]^ Excludes participants in later waves who were not eligible to distribute coupons once the study approached its target sample size;

^[c]^ Excludes pre-testers (n = 17) and duplicates (n = 2);

^[d]^ Includes additional 8 coupons distributed by 6 individuals with missing enrollment data;

^[e]^ Excludes n = 23 productive and unproductive seeds who did not return with coupons because of seed status.

**Fig 2:**
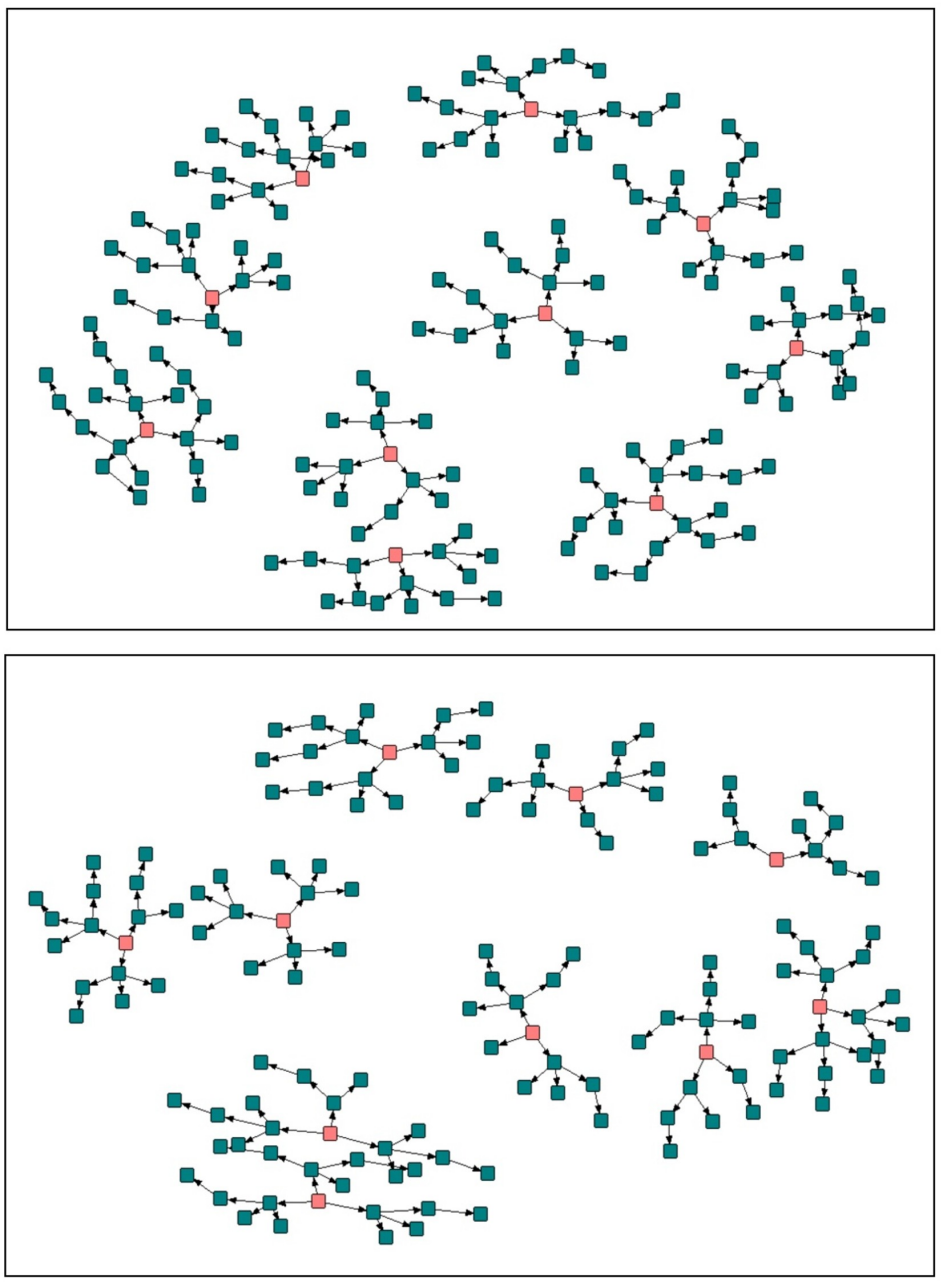
Recruitment network of study participants in Korogocho (top figure) and Kawangwere (bottom figure) with twenty seeds indicated by orange squares.

Fig 2 provides a pictorial diagram of each urban slum settlement’s RDS network. The mean (SD) reported network size of peers known by the participants who were aged 18–22, Kenyan, and living in their urban slum settlement was 19.0 (±30.2). On average, participants in Kawangwere reported larger peer networks of 21.6 (±34.6) than those in Korogocho (16.4, ±24.9). Approximately 9% of participants were unable to quantify their networks, although the majority were able to quantify individuals who they knew (Table 1). The majority (74%) of participants described their recruiter as a “friend” compared with 9% and 14% who described their recruiter as a relative or neighbor, respectively (Table 1). The average number of years knowing one’s recruiter was 7.3 (±7.4). Young women in Korogocho appeared more likely than young men to have a recruiter they did not know (5% vs. 3%). In Kawangwere, young women also appeared more likely than young men to be recruited by a relative or neighbor (16% and 13% vs. 5% and 10%, respectively). Young men in Kawangwere had the highest recruitment by their friends (84%). Most participants (83%) reported meeting their recruiter in the urban slum settlement compared to meeting them at school (6%), at their job (1%), at a religious gathering (1%), or elsewhere (9%), such as a community center or unknown. Nearly all (99%) of participants positively described the nature of their recruiter’s invitation, having been reminded on average 1.9 times (±1.2) to return to the study site. The study team considered five or more reminders as excessive. Ten participants (n = 10, 3%) reported being reminded 5 or more times by their recruiter.

**Fig 3:**
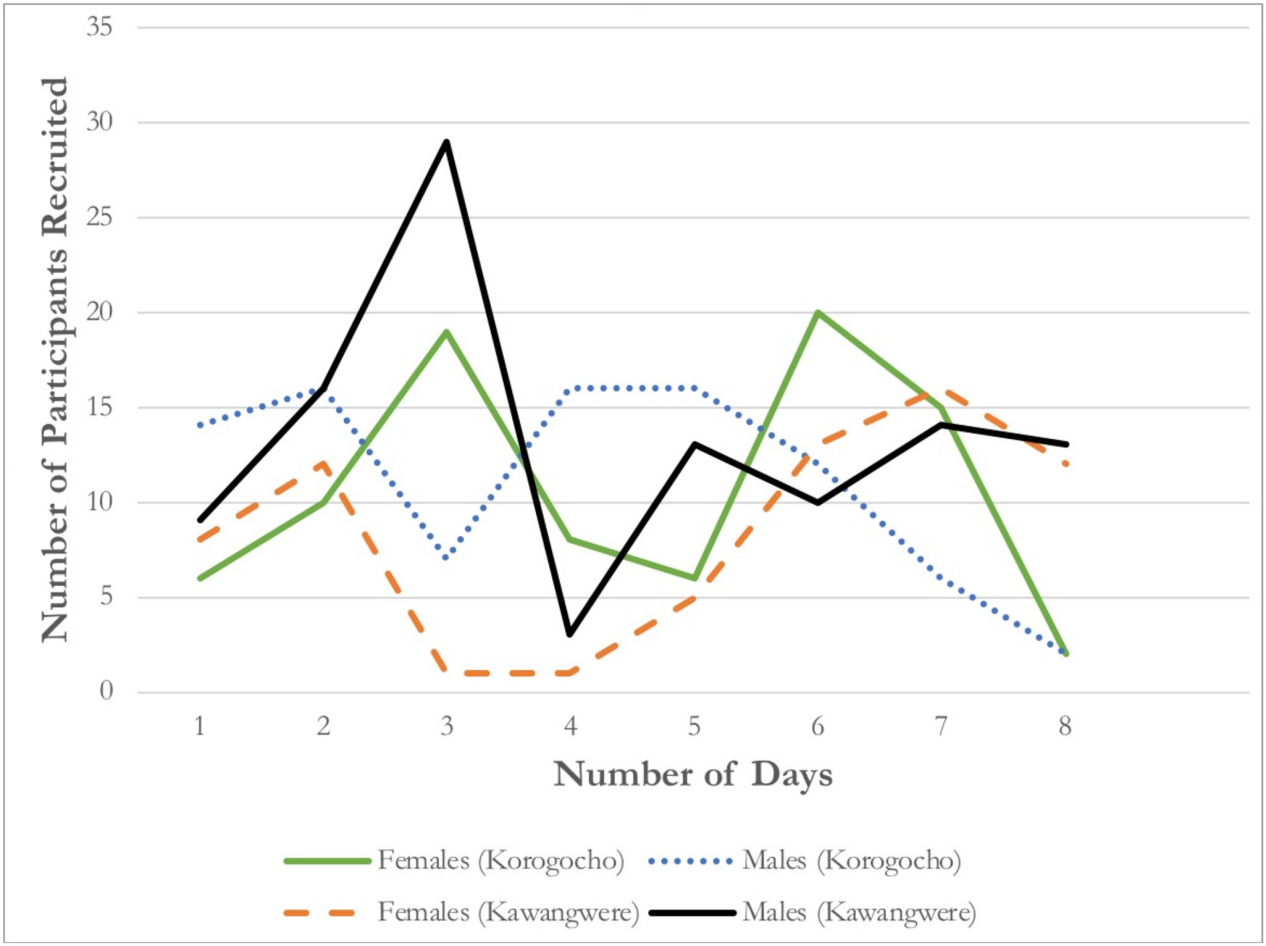
Daily recruitment by gender and by site over the study period.

**Fig 4:**
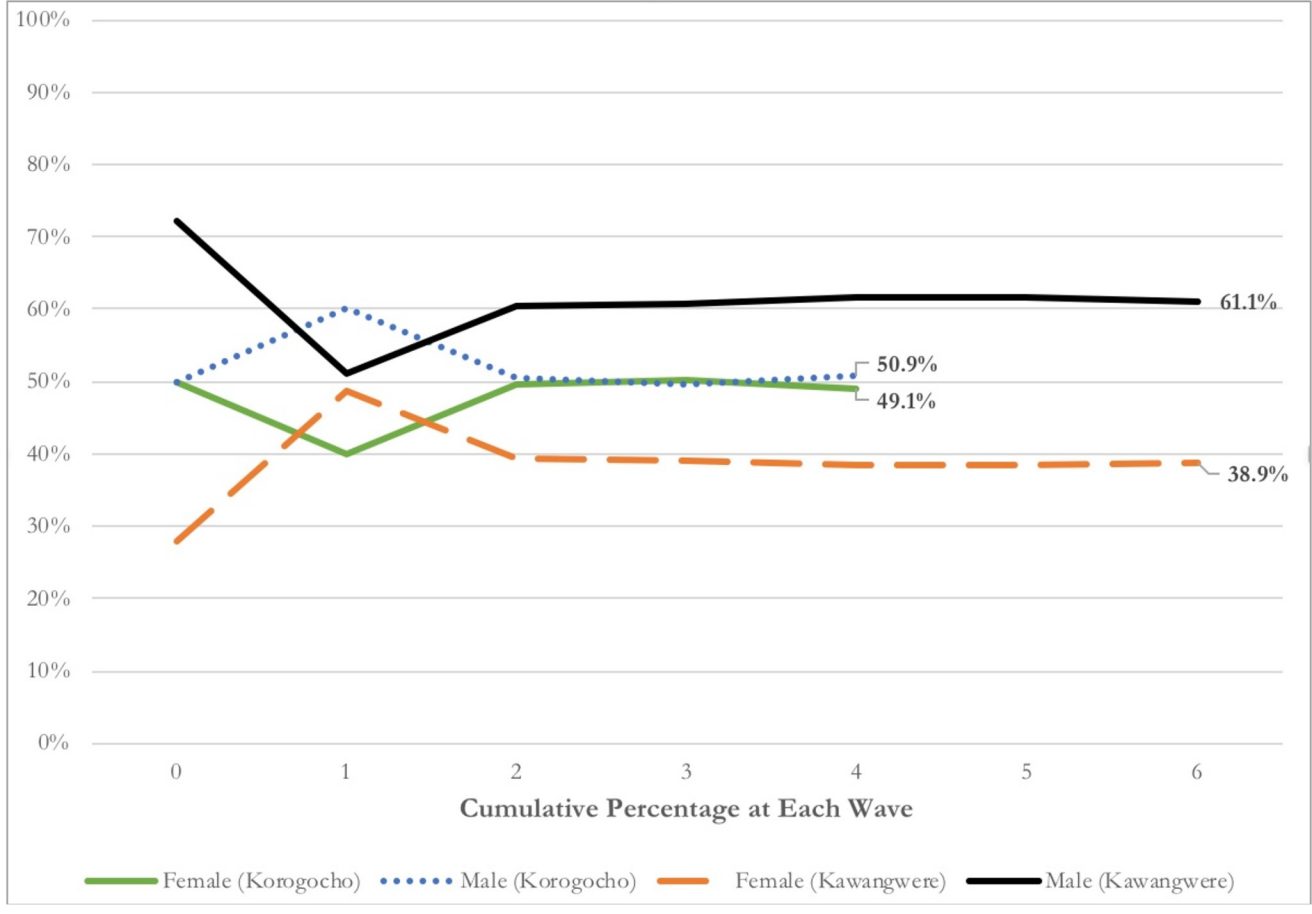
Full sample equilibrium by study site, gender, and recruitment wave.

The study recruited participants for 8 days (excluding weekends), resulting in an average of 39.0 (±16.7) recruits each day (~20 per day per site). Fig 3 depicts the number of participants recruited per day by site and by gender. In the first 3 days of the study, young women were consistently less likely to be recruited compared to young men. However, the number of female recruits peaked towards the end of the recruitment period (Fig 3). Over time, an equal cumulative percentage of male and female recruits was achieved in Korogocho (49% young women; 51% young men) as shown in Fig 4. However, the cumulative percentage of young women at the end of the study in Kawangwere was 39% compared to 61% young men (Fig 4). Equilibrium, in which gender proportion remained static and did not fluctuate by more than 2% thereafter, was achieved in both sites by wave 2 (Fig 4). On average, a maximum of 3.0 weekdays were needed for all of the recruiter’s recruits to return for survey payment (Table 1). As the study approached its target sample size, we reduced the number of coupons distributed from 3 to 1 to 0 (Table 1). Fifty-six percent (56%, n = 197) of participants were eligible to receive coupons compared to 44% (n = 153) who were not invited to continue the recruitment chain. Of those eligible, 96% (n = 189) were willing to distribute coupons. Twenty-three percent (23%, n = 80) of the total sample received three coupons compared to 33% (n = 117) and 44% (n = 153) who received 1 or 0 coupons, respectively. Willingness to distribute coupons was rated an average of 8.7 (a score of 10 being the maximum level of willingness) (Table 1). A total of 365 coupons were distributed and 327 coupons were returned, representing a 90% coupon return rate (identification rate). This indicates that the majority of participants successfully recruited someone. The coupon return rate were higher in Korogocho (93% coupons returned per distributed) compared to 87% in Kawangwere. The 327 coupons returned did not include the 20 productive and 3 unproductive seeds that were enrolled in the study (totaling n = 350). Two participants (n = 2, 0.6%) reported safety concerns as a result of study participation and were referred to and monitored by the study supervisor. The screening threshold was 1.0 in that it appears ineligible participants were not identified by peers or self-selected out of the study prior to arrival.

### Distribution of demographic and sexual risk behaviors

The distribution of demographic and sexual risk behaviors among participants is shown in Table 2. The mean age of participants was 19.4 (±1.3, weighted 19). RDS overcame feasibility concerns of household-, clinic-, and school-based sampling methodologies in that underserved young adults who were unemployed in the last 7 days (64%, weighted 68%), unemployed in the last 6 months (31%, weighted 33%), not currently enrolled in school (48%, weighted 47%), identifying as an ethnic/religious minority (25%, weighted 26%), and having prior residential instability (≥2 moves in the past year, 19%, weighted 19) were successfully recruited (Table 2). Youth reporting sexual risk behaviors, including prior unprotected sex (34%, weighted 38%), sex while high/drunk (37%, weighted 34%), sex exchange for pay (12%, weighted 14%), and sex with 3 or more sexual partners (13%, weighted 13%) were also enrolled (Table 2). Seventy-eight percent (78%, n = 180, 77% weighted) of sexually-debuted participants (n = 231, 66%, 61% weighted) had engaged in at least one of these four sexual risk behaviors. However, 34% of participants (n = 119, 39% weighted) had not sexually debuted.

**Table 2:** Crude and respondent-driven sampling (RDS)-adjusted prevalence estimates of demographic and sexual risk behaviors (N = 350).

*Demographic Characteristics*	Crude		RDS-weighted	
	N	%^a^	%^a^	(95% CI)
Mean age (in years) (±SD)	19 (±1.3)	-	19	(19, 19)
Highest level of education completed				
Never attended school	3	1	1	(0, 2)
Primary	172	49	52	(45, 59)
Secondary or higher	174	50	47	(40, 54)
Not currently enrolled in school	175	48	47	(40, 53)
Mean # years living in urban settlement (±SD)	14 (±6.7)	-	13	(12, 14)
# of times moved dwelling within last year				
0	222	63	63	(56, 69)
1	59	17	17	(13, 23)
2 or more	67	19	19	(14, 25)
Marital status				
Single	323	92	92	(87, 95)
Married/Cohabitating	19	5	6	(3, 10)
Widowed	1	0	0	(0, 1)
Separated/Divorced	6	2	2	(1, 6)
Tribal Ethnicity				
Luo	22	6	5	(3, 9)
Luhya	43	12	13	(9, 19)
Kikuyu	163	47	46	(39, 53)
Garre^c^	40	11	12	(8, 18)
Borana^c^	18	5	5	(3, 9)
Mixed	6	2	2	(1, 6)
Other	57	16	16	(12, 22)
Religious Affiliation				
Christian	257	73	73	(66, 78)
Muslim^c^	89	25	26	(21, 33)
Other	1	0	1	(0, 5)
None	3	1	0	(0, 1)
Mean # of people in household, including self (±SD)	5 (±2.6)	-	5	(5, 5)
Unemployed in last 6 months	107	31	33	(27, 40)
Unemployed in last 7 days	223	64	68	(62, 74)
*Sexual Behaviors*				
Among all participants (n = 350)				
Ever had sex	231	66	61	(54, 67)
Among sexual-debuted participants (n = 231)				
Had sex in last 6 months	153	66	67	(59, 75)
Had unprotected sex at last sex^c^	78	34	38	(30, 47)
Had sex while high/drunk in the last 6 months	86	37	34	(26, 42)
Had sex in exchange for money, food, or housing in last 6 months	28	12	14	(9, 21)
Had sex with 3 or more sexual partners in last 6 months	30	13	13	(8, 20)
Reported at least one sexual risk behavior in last 6 months^d^	180	78	77	(69, 83)

^[a]^ Percentages not adding to 100 are due to missing or don’t know responses.

^[b]^ Excludes participants who have never had sex;

^[c]^ Religious/ethnic minorities;

^[d]^ Includes unprotected sex, sex while high/drunk, sex exchange, and sex with 3 or more sex partners.

One hundred seventy-two (n = 172, 49%, weighted 52%) of the sample had primary education (~ 8 years of schooling) as their highest level of education compared to 174 participants (50%, weighted 47%) who reported having secondary or higher education. The average reported number of years living in their settlement was 14.1 (±6.7, weighted 13.1) years. Most recruits (n = 323, 92%, weighted 92%) were unmarried and not cohabitating with a sexual partner. The sample was ethnically diverse: 47% (weighted 46%) Kikuyu, 12% (weighted 13%) Luhya, 11% (weighted 12%) Garre, 6% (weighted 5%) Luo, 5% (weighted 5%) Borana, and 18% (weighted 18%) mixed or other. The reported number of people living in each participant’s household (including self) was 4.9 (±2.6, weighted 4.9). Of the 3 unproductive seeds: 33% were male; 33% were unemployed in the last 7 days; 33% were unemployed in the last 6 months; 33% unstably housed; 67% were out-of-school and 33% reported at least one sexual risk behavior. Of the 3 replacement seeds: 100% were male; 0% were unemployed in the last 7 days; 0% were unemployed in the last 6 months; 0% unstably housed; 33% were out-of-school and 0% reported at least one sexual risk behavior.

### RDS successes and challenges

Table 3 summarizes successes, challenges, and lessons learned for several aspects of the RDS methodology. Key successes included having productive seeds, including successful replacement of non-productive seeds, achieving a rapid recruitment pace (20 to 30 participants per site per day), using study-stamped coupons to reduce misuse, using sufficiently broad inclusion criteria, motivating participants through small payments and task empowerment, as well as achieving a sample of participants with diverse demographic characteristics. The sampling approach was also found to be safe with rare reported negative consequences. RDS challenges included managing the large number of participants visiting the study site, including wait times, continuing community engagement to encourage enrollment of young women, and discouraging demands by some recruiters of non-study payment from their recruits. Use of community representatives, incentives, and discussions of ways to improve guidance to recruiters and recruits (such as on estimating network size, announcing coupon cessation, requesting payment only from the study team, and limiting the number of reminders) were important lessons learned.

**Table 3:** Summary of RDS successes, challenges, and lessons learned.

RDS Process Areas	Success	Challenges	Lessons Learned
Seed Productivity	Most seeds were productive and yielded recruits who also recruited. Males were more likely to be productive seeds.	Two female unproductive seeds and one male unproductive seed were replaced by three additional young men.	Community liaisons were resourceful in identifying study seeds. Support to reach out to lost seeds and understand reasons for unproductivity is needed.
Recruitment Pace	Recruitment pace was rapid with 20 to 30 participants visiting the study site daily. Some recruits returned in the same day. Reminders from peers may have contributed to the rapid pace.	At peak, participants had longer wait times. And, as the study approached its target sample, participants were disappointed when coupon disbursements stopped.	Reducing the number of coupons distributed and lengthening the coupon expiration time managed the pace. Other incentives may be needed for end-sample participants who cannot recruit.
	Study staff were trained and on-site from morning to evening with rotating lunch shifts.	Study staff were subject at times to transportation delays to urban slum settlements on the outer edge of the city.	Transportation, lodging, and/or meal support to study staff may be needed.
Ineligibility & Coupon Misuse	All study coupons and stubs were stamped with a unique sticker seal to reduce the production of counterfeit coupons.	Coupons were paper-based and were vulnerable to being lost, stolen, or damaged although there were no reports of this.	Including a study seal on RDS coupons was an effective strategy.
	Eligibility criteria were relatively broad and assessed by the field supervisor. All presenting youth were eligible for participation resulting in a low screening threshold.	Eligibility was determined based on self-report. Government IDs to confirm birth date or residence were not used so as to minimize barriers to participation.	Eligibility criteria were simple enough that recruiters pre-screened peers. Potential peers were screened again by study staff and confirmed against a list of previous enrollees’ first names and birth dates.
Incentive Motivation	Youth valued payments for survey completion and for successful recruits. Recruiting was sometimes viewed as positive short-term job, which may have further motivated participation.	Some recruits reported being asked by recruiters to remit a portion of their survey payment to the recruiter. Some older youth who were study-ineligible also requested payment for protecting the study site.	Small incentives were an effective strategy to boost recruitment. More guidance to recruiters regarding behavioral expectations may be helpful.
Network Diversity	Sampled youth are diverse with regards to age, school enrollment, gender, ethnicity, and religion. Participants also included high-risk individuals and sex workers.	Some households were concerned and/or reluctant to allow young women to leave their homes to participate in the study. The proportion of young women was low in one site.	Community representatives were critical for initial and on-going communication regarding study purpose and activities. Youth recruit eligible peers who are like and unlike themselves.
Applicability of RDS Assumptions	Most recruiters knew their recruits and selected their friends. Recruiters may also have selected friends perceived as more likely to respond.	9% recruits had difficulty reporting the number of individuals they knew and relied on guestimates or reported as uncountable.	Assistance in accurately counting peer network size may be needed for participants if inclusion criteria are broad.
	Two duplicates was identified despite the study’s assumption of sampling with replacement.	Some potential recruits may have self-selected out of being recruited again, resulting in sampling without replacement.	Guidance to participants on how to randomly select the requested number of peers out of all known eligible peers may strengthen random selection.

## Discussion

Respondent-driven sampling (RDS) is an effective tool for recruiting underserved, high-risk groups that cannot be randomly sampled. Our study found that a large and diverse sample of eligible young adults could be achieved in a relatively short period of time. While RDS has been used previously to recruit several populations at risk for HIV such as MSM and FSW, our study is among the first to use RDS in an impoverished urban slum settlement to identify young adults experiencing structural risk factors of HIV, such as unemployment, low income, and residential instability, and may represent generalizable findings for this target population. At relatively low cost, the peer-to-peer approach enabled the study to reach young adults with varying HIV prevention needs who may have been overlooked by household, clinic-, or school-based sampling strategies.

These findings point to several lessons learned regarding the potential of RDS and opportunities to maximize its effectiveness in biological or behavioral HIV research. One lesson learned relates to ability to recruit and to peer network size. An advantageous finding was that the majority of paricipants successfully recruited someone and reported having a relationship with their recruiter. In addition, most participants were able to quantify a relatively large peer network. Large peer networks have been shown in some studies to be correlated with high numbers of sexual partners and subsequent HIV risk [20]. Participants’ reportedly large networks of individuals who they knew may have reflected, in part, the study’s broad inclusion criteria which did not base eligibility on sexual orientation, sex work, or other potentially stigmatizing factors. While living in an “urban slum settlement” may be stigmatizing in wealthier settings, young adults recruiting within these settlements shared this characteristic. The study’s inclusion criteria on age, residence, and nationality were also easy to verify by recruiters who may have been unable to estimate network sizes of peers engaged in specific sexual risk behaviors. For example, while young adults may have been aware of peers who were MSM or FSW, they may have lacked knowledge of those non-MSM/non-FSW who were also at risk to HIV as a result of unsafe and unprotected sexual practices. Having readily-verifiable inclusion criteria meant also that individuals who were ineligible (i.e., under-age, not residing the settlement, not Kenyan) were likely to have self-selected out of the study prior to any screening, either at the time of invitation or when informed of the study’s purpose. In addition to facilitating large network sizes, this resulted in the study’s having high efficiency and a high screening threshold.

Conversely, having large peer networks presented a challenge for a few participants. An assumption of RDS is that participants are able to provide accurate measures of the size of eligible peers they know by name and who know them. However a small proportion of participants in this study had difficulty providing a precise number and responded by saying “very many” or “uncountable”. Although uncommon, more assistance may be needed to assist young adults in counting particularly large networks. For example, because most young adults reported that their recruiters were “friends”, linking the network size to the sum of their reported number of “friends” on social media or in other real or virtual social groups may help participants estimate a more accurate number. Another strategy may be to ask participants to count eligible peers who they know and with whom they have had recent contact over a series of ascending number of weeks or months or to count eligible peers who they are likely to see within a certain period in the future.

A second lesson learned relates to the pace of recruitment in achieving the target sample size in a relatively short period of time. Several factors may have contributed to the successful pace. Data collection was conducted within the urban settlement, thereby reducing the need for extensive travel and costs by potential participants. The printed recruiting instructions provided to participants on the coupon was simple and easy to follow. This enabled potential recruiters to quickly understand the assigned task. In addition, three or more interviewers were present at the study site throughout the day to conduct interviews for the convenience of presenting recruits, who were then readily screened, and, if eligible, enrolled in the study.

A third lesson learned relates to using RDS to recruit impoverished young women. Much of the evidence for RDS in HIV prevention research has involved gender-stratified studies involving only men, only women, or only gender-transitioning individuals [25–29, 31–34, 45]. Our study provides insight into potential gender differences in RDS implementation. Young women had higher reports of being recruited by someone they did not know and fewer reports of being recruited by a friend. They also had consistently lower enrollment than young men at the beginning of the study and were not equally represented (50% of the total sample). Having fewer productive female seeds in one site may have contributed to the imbalance. While our study observed that several of our recruitment approaches enhanced the participation of young women, such as using study sites within and walkable from each of the urban slum settlements, recruiting based upon non-stigmatizing characteristics, having female seeds, avoiding study activities at night when violence is more common, and allowing participants to arrive and wait at study sites in groups (which many young women did), some barriers to participation may have remained. These barriers could have been that young women’s networks were simply less accessible than young men’s. For example, at the start of recruitment, a community representative indicated that fewer young women were coming to the study site because some parents were reluctant to allow them to leave their homes due to rumors that the study resulted in young women being absent, and unaccounted for, for several hours. We relied on on-going communication by community representatives to explain the study’s purpose and short survey duration, which appeared to increase the numbers of female recruits towards the end of the study. Young women may also have recruited more slowly as a result of having caretaking or employment responsibilities or due to being married and having less available time for study participation. Given these constraints, young women may also have been less motivated by our financial incentive. Future RDS studies may benefit from using larger incentives for survey completion, enrolling more female seeds, or providing higher pay for each successful female recruit. Encouraging young men to recruit female peers or distributing a greater number of coupons to female recruiters may also be beneficial.

A fourth important finding was that RDS appeared to reach into urban slum networks that may have been underrepresented in some studies that used traditional sampling methods. This was a particularly positive outcome of our study given the under-representation of some high-risk groups in current surveillance samples [11–14]. In fact, the inclusion of several Kenyan ethnic minorities (i.e., Garre/Borana) was an unexpected finding. However, lower mean age (19 years old) and inclusion of some young adults with no prior sexual history suggested that the survey also tapped into networks of lower risk individuals that could also bias estimates of behavioral prevalences. While the study benefited from penetrating into a diverse network of young adults, future RDS efforts should consider the trade-offs of using a wide-net versus more targeted enrollment strategy of sub-groups most at-risk. Questions remain as to whether RDS can as successfully rely on young adults to recruit peers who are engaging in a range of sexual risk behaviors or who are exposed to similar socio-economic factors underlying HIV risk. In this study, our analysis of RDS-recruited young adults indicated a high level of sexual activity and sexual risk-taking in this population.

Finally, the study gained knowledge in the use of monetary incentives for RDS. The 50 Kenyan shillings provided as payment for each enrolled recruit was sufficient to cover one day’s public transportation costs or purchase a small lunch. Therefore, the study team felt that the incentive amount was small enough to be non-coercive, but large enough to motivate participation. The high rate of returned coupons suggests also that incentives were a motivating factor—and that young adults who were predominately unemployed were attracted to the idea of being tasked and very shortly “employed” with recruiting responsibilities. They may also have valued working with local study personnel. However, in a small number of cases, recruiters appeared to be overly enthusiastic given that the study had some recruits reporting “no relationship” to their recruiters. It is possible that these recruiters were motivated to be paid, but unable to identify a peer who was eligible, willing, and available to travel to the study site within the allowable timeframe. On these rare occasions, we did not exclude coupon-holding recruits from unknown recruiters given our inability to re-equip recruiters with more coupons as well as our desire to continue assessing the RDS chain. However, recruiters in later waves were reminded to select individuals they knew who were also residents of the urban slum settlement. This was the core element that created the network and resulted in overlapping social and sexual networks. Another lesson learned relating to RDS incentives was that some recruits reported that their recruiter asked for a portion of the survey payment as remittance for study referral. Although in the majority of the cases, incentives were a positive motivation, additional communication during enrollment regarding payment expectations between recruiters and recruits would be useful. This includes inviting early recruiters to inform recruits that they may not, in turn, be eligible to recruit (and receive coupons and recruiter payments) if the study is in the later stages of enrollment. As word spread, some recruits in later waves were disappointed not to similarly be given recruiting responsibilities, interact with their peers, and earn income for study referrals.

More research is needed on the use of incentives for RDS participants in urban slum settlements. While effective for most, the lower participation of women and the occurrence of three unproductive seeds (out of 20) suggests that additional types of incentives or supports are needed. For example, using phone calls or text messages to follow-up with unproductive seeds to remind them to recruit peers or to assess reasons for loss-to-follow-up would help improve the RDS process. Obtaining feedback also on number of and reasons for peer refusals would guide our understanding of how best to align incentives with barriers to participation. Given our unexpectedly high coupon return rate, starting with fewer seeds or fewer coupons per participant may also have yielded more waves. It is promising nonetheless that so many young adults responded favorably and rapidly to the peer-to-peer sampling approach.

### Limitations

The study’s limitations should be taken into account. This study relied on a descriptive process evaluation to assess the feasibility of the RDS methodology. While RDS facilitated identification of underserved young adults who may have been missed by other sampling strategies, a comparative statistical analysis of these methodologies is needed to determine relative effectiveness. With larger sample power, the gender differences we observed in the outcomes of RDS should also be comparatively analyzed. As mentioned previously, efforts are needed to improve participant’s ability to report peer network size. While several participants reported a number of known eligible peers which we used to estimate selection probabilities, responses may not have been precise. We also do not know the extent to which other biases unrelated to network size may have been in effect. While the study achieved a diverse demographic sample, no geocoding data were used to assess geographical spread, and conducting the study during weekdays may have limited participation of young adults who were at work/school or otherwise unable to travel to the study site. To minimize barriers to participation, we did not require finger prints, proof of residency, or government-issued identification to confirm study eligibility, as many young adults in Kenya do not have them. Site managers instead compared the first names and birth dates of all recruits to a list of prior study enrollees. It is therefore possible that some unidentified duplicates or young adults living in other urban slum settlements were included. However, despite these limitations, the study had several strengths. It is among the first to present findings regarding the structure of social networks of young adults living in sub-Saharan African urban slum settlements. We also provide several quantifiable process measures and insights into effective strategies for recruiting underserved and at-risk young adults.

## Conclusion

Less is known regarding the effectiveness of using RDS to recruit for and assess sexual risk behaviors in young adults living in high HIV-prevalence urban slum environments. Our findings indicate that RDS should not be underestimated as it may be an important tool for increasing participation and representation of this population in bio-behavioral research. Reasons for our success are likely due to the use of monetary incentives, community representatives, simple inclusion criteria, coupon expiration dates, and convenient study sites. As a result, recruiters rapidly identified diverse and eligible peers. RDS improvements in the future for urban slum young adults should focus on measuring network size, enhancing recruiter-recruit communication, and using RDS for more targeted referrals based on a range of underlying structural and behavioral risks to HIV.

## Supporting information

S1 Questionnaire (PDF)

S2 Questionnaire (PDF)

## Abbreviations

CI: Confidence interval
DHS: Demographic and Health Survey
FSW: Female sex worker
HIV: Human Immunodeficiency Virus
ID: Identification number
MSM: Men who have sex with men
NAHEDO: National Health and Development Organization
RDS: Respondent-driven sampling
USD: United States dollar
