# Supplementary material for: Lessons learned from using respondent-driven sampling (RDS) to assess sexual risk behaviors among Kenyan young adults living in urban slum settlements: A process evaluation
Source: PLoS One. 2020 Apr 10;15(4):e0231248. doi: 10.1371/journal.pone.0231248 (PMC7147752; doi:10.1371/journal.pone.0231248)
Supplement: S1 Questionnaire — (PDF) [file pone.0231248.s001.pdf]

ID: ___________-___
Date: ___/____/_____

Study Title: Economic context and HIV vulnerability in young adults living in urban slums in Kenya
SURVEY QUESTIONNAIRE
Young Adults, Aged 18 to 22 Years

COMPLETE BEFORE AND AFTER THE INTERVIEW:	
**01 Participant ID**	|___|___|___|___|
**02 INTERVIEWER ID**	|___|___|
**03 INTERVIEW DATE**	_____/______/_______<br>Day Month Year
**04 INTERVIEW DAY**	1 – Monday 2 – Tuesday 3 – Wednesday 4 – Thursday 5 – Friday 6 – Saturday 7 - Sunday
**05 START TIME**	_____:_____<br>Hour Minute
**06 END TIME**	_____:_____<br>Hour Minute
**07 INTERVIEW DURATION**	_______ Less or equal to 90 minutes _______ More than 90 minutes
**08 LOCATION**	1 = Korogocho 2 = Kawangware (Select one)
**09 LANGUAGE**	1 = English 2 = Kiswahili (Select one)

Section 1: Demographic Characteristics
Before we get started, I would like to explain to you how the interview works. As the screener described to you, your participation in the interview and every aspect of the study are completely voluntary. You may skip any questions that you prefer not to answer, but we would appreciate your cooperation. You may also ask me to clarify questions if you don't understand them or decide to stop the interview at any time. Finally, all the information that you provide for the study is kept completely confidential. Your responses to our questions are identified only by number, never by name. I would like to start by asking a few general questions.

No.	Question	Response	Skip
1.0A	**What is your relationship to the person who gave you the study's recruitment coupon? (Select only one)**	01 Friend<br>02 Family member<br>03 Neighbor<br>04 No relationship<br>05 Other (specify): ______________________<br>88 DK 99 NR 77 Not-Applicable (NA)	
1.0B	**How many years have you known this person?**	|___||___| years old 88 DK 99 NR 77NA	
1.0C	**Where did you first meet the person who recruited you?**	01 In community<br>02 At school<br>03 At work or job<br>04 At religious gathering<br>05 Other (specify): ______________________<br>88 DK 99 NR	
1.0D	**How many times did this person remind you to participate in the study?**	|___||___| Times 88 DK 99 NR	
1.0E	**How would you describe the nature of the invitation from your recruit?** [Select all that apply]	01 Friendly<br>02 Aggressive (Pushy)<br>03 Exciting<br>04 Worrisome<br>05 Other (specify): ______________________<br>88 DK 99 NR	
1.1	**Gender**	01 Male<br>02 Female<br>03 Other (specify): ______________________	

ID: __________-___
Date: ___/____/_____

Section 1: Demographic Characteristics<br>*Before we get started, I would like to explain to you how the interview works. As the screener described to you, your participation in the interview and every aspect of the study are completely voluntary. You may skip any questions that you prefer not to answer, but we would appreciate your cooperation. You may also ask me to clarify questions if you don't understand them or decide to stop the interview at any time. Finally, all the information that you provide for the study is kept completely confidential. Your responses to our questions are identified only by number, never by name. I would like to start by asking a few general questions.*			
**No.**	**Question**	**Response**	**Skip**
1.2	**How old are you?**	|___||___| years old 88 DK 99 NR	
1.3	**What is the highest level of education you have completed?**	00 Have never attended school →<br>01 Primary<br>02 Secondary or higher<br>88 DK 99 NR	
1.4	**Are you currently enrolled in school?**	01 Yes 02 No 88 DK 99 NR	
1.5	**How long have you been living in this settlement?**	[___|___] Years [___|___] Months<br>88 DK 99 NR	
1.6A	**How many young adults (aged 18 and older) are there that you know and that know you, and that live in this urban settlement (Korogocho or Kawangware)?**	[___|___][___|___]Number of adults, all ages<br>88 DK 99 NR	
1.6B	**Of them, how many are aged 18 to 22?**	[___|___][___|___]Number of adults, aged 18-22<br>88 DK 99 NR	
1.7	**How many times have you moved dwellings within the last year?**<br>NOTE: Either within or outside of the settlement.	[___|___] number of times moved<br>88 DK 99 NR	
1.8	**What is your current marital status?**	01 Married or Cohabiting<br>02 Widowed<br>03 Divorced/Separated<br>04 Single 88 DK 99 NR	
1.9	**What is your triabal ethnicity?**	01 Luo<br>02 Luhya<br>03 Kikuyu<br>04 Other (specify): ________________	
1.10	**What is your primary religious affiliation?**	01 Christian<br>02 Muslim<br>03 No religious affiliation<br>04 Other (specify): ____________________	
1.11	**How many people at the moment live in your house including yourself?**	|___||___| number of people<br>88 DK 99 NR	

Section 2: Earned Income<br>*I will now ask you about your sources of income whether it be through work that you do for others or for yourself.*			
**No.**	**Question**	**Response**	**Skip**
2.1	**In the last 6 months, did you perform any activity for pay, profit, or barter?**	01 Yes 02 No 88 DK 99 NR	
2.2	**In the last 7 days, did you perform any activity for pay, profit, or barter?**	01 Yes 02 No 88 DK 99 NR	
2.3	**How many weeks or months ago did you last do any work for pay, profit, or barter?**	[___|___] Years [___|___] Months<br>88 DK 99 NR	
2.4	**On an average week day, how many hours do you spend he/she spend working and earning money or goods?**	[___|___] Hours<br>88 DK 99 NR	

**Section 2: Earned Income**<br>*I will now ask you about your sources of income whether it be through work that you do for others or for yourself.*			
**No.**	**Question**	**Response**	**Skip**
2.5	**On an average weekend-day, how many hours do you spend he/she spend working and earning money or goods?**	[___|___] Hours<br><br>88 DK 99 NR	
2.6	**Have you operated any business(es) or have done any self-employed activity during the last year?**	01 Yes 02 No 88 DK 99 NR	
2.7	**What kind of business(es) do you operate? (Select all that apply)**	01 Driver, Transportation<br>02 Vendor (selling items)<br>03 Service (food, clothing)<br>04 Entertainment<br>05 Domestic care<br>06 Other (specify):____________________	
2.8	**What was your net income (profit) from your business(es) on average, during a one month period?**<br>(Select only one)	KSH 25,000 OR LESS……………01<br>KSH 25,001 – 50,000……………..02<br>KSH 50,001 – 75,000……………..03<br>KSH 75,001 – 100,000……………04<br>KSH 100,001 – 150,000………....05<br>KSH 150.001 – 200,000…………..06<br>KSH 200,001 – 300,000………….07<br>KSH 300,001 – 400,000…………..08<br>KSH 400,001 – 500,000…………..09<br>KSH 500,001 OR MORE…………10<br>88 DK 99 NR	
2.9	**Did you do any wage work during the last year?**<br>NOTE: Remember that by wage work, I mean work for someone else for pay. You may have been paid in cash or another form or payment.	01 Yes 02 No→ 88 DK→ 99 NR→	
2.10	**What kind of wage work have you done in the last year?**<br>NOTE: If you have more than one wage job, select your primary wage job.	01 Driver, Transportation<br>02 Vendor (selling items)<br>03 Service (food, clothing)<br>04 Entertainment<br>05 Domestic care<br>06 Other (specify):____________________	
2.11	**Thinking of all the wage work you have done, how much cash did you earn from these activities, on average, during a one month period in the last year?**<br>**(Select only one)**	KSH 25,000 OR LESS……………01<br>KSH 25,001 – 50,000……………..02<br>KSH 50,001 – 75,000……………..03<br>KSH 75,001 – 100,000……………04<br>KSH 100,001 – 150,000………....05<br>KSH 150.001 – 200,000…………..06<br>KSH 200,001 – 300,000………….07<br>KSH 300,001 – 400,000…………..08<br>KSH 400,001 – 500,000…………..09<br>KSH 500,001 OR MORE…………10<br>88 DK 99 NR	
2.12	**Would you say that you are currently employed?**	01 Yes 02 No→ 88 DK→ 99 NR→	
2.13	**Which of the following best describes your current employment situation?**<br>**(Select one)**	01 Full-time<br>02 Part-time<br>03 Self-employed (informal sector)<br>04 Unemployed<br>05 Other (specify): _____________________<br>88 DK 99 NR	

Section 2: Earned Income<br>*I will now ask you about your sources of income whether it be through work that you do for others or for yourself.*			
**No.**	**Question**	**Response**	**Skip**
2.14	**Over the past 6 months, where did you get most of your money?**<br>**(Select one)**	01 Self-owned business<br>02 Wage work (paid by others)<br>03 Grants (child support, disability)<br>04 Cash gifts from sex partner<br>05 Cash gifts from family or friends<br>06 Sex work<br>07 Did not have any money<br>08 Other (specify): ____________________<br>88 DK 99 NR	
2.15A	**In Kenyan Shilings, what is your average individual monthly income?**	|___||___| |___||___|ksh 88 DK 99 NR	
2.15B	**What would be your ideal average individual monthly income?**	|___||___| |___||___|ksh 88 DK 99 NR	
2.16	**Who usually decides how the money you earn will be used?**<br>**(Select one)**	01 Respondent<br>02 Jointly with sexual partner<br>03 Sexual partner only<br>04 Joint with another person<br>05 Another person only<br>88 DK 99 NR	
2.17	**In Kenyan Shilings, how much money is given to you by family or friends in any given month?**	|___||___| |___||___|ksh 88 DK 99 NR	
2.18	**In the past 6 months, how has your income changed? (i.e., increased, decreased or stayed the same)**	01 Increased 03 Stayed the Same→<br>02 Decreased 99 NR→	
2.19	**How much did your income change from the past month?**	|___||___| |___||___| ksh 88 DK 99 NR	
2.20	**Do you currently have a banking account?**	01 Yes 02 No 88 DK 99 NR	

Section 3: Savings and Debt			
3.1	**Are you a member of a savings groups?**	01 Yes 02 No 88 DK 99 NR	
3.2	**Have you saved any money within the past 12 months?** NOTE: This includes any cash or assets put aside personally or in a bank.	01 Yes 02 No 88 DK 99 NR	
3.3	**Have you set money aside for savings within the last 6 months?** NOTE: This includes any cash or assets put aside personally or in a bank.	01 Yes 02 No 88 DK 99 NR	
3.4	**What is the total amount of money you have currently saved? (Answer in Kenyan Shillings)**	|___||___| |___||___| ksh 88 DK 99 NR	
3.5	**Do you have any money that you borrowed and will have to repay to a person or group of people?**	01 Yes 02 No 88 DK 99 NR	
3.6	**How much debt do you owe?**	|___||___| |___||___| ksh 88 DK 99 NR	
3.7	**How much money do other individuals currently owe you?**	|___||___| |___||___| ksh 88 DK 99 NR	
3.8	**Over the past 12 months, have you borrowed cash from an institution?**	01 Yes 02 No→ 88 DK 99 NR	
3.9	**How much was borrowed?**	|___||___| |___||___|___||___| ksh<br>88 DK 99 NR	

3.10	**What did you use your borrowed cash for?**	01 Start a business<br>02 Household expenses<br>03 Medical expenses<br>04 Educational expenses<br>05 Other (specify): _______________<br>88 DK 99 NR	
3.11	**Over the past 12 months, have you borrowed money from a friend, partner, or relative?**	01 Yes 02 No 88 DK 99 NR	
3.12	**What is the total amount that you owe for repayment of all outstanding debts?**	|___||___| |___||___| ___||___|ksh<br>88 DK 99 NR	
3.13	**If you needed to obtain a loan today for 5,000 KSH (about $50 USD), would you be able to do it?**	01 Yes 02 No 888 DK 99 NR	
3.14	**If you needed to obtain a loan today for 10,000 KSH (about $100 USD), would you be able to do it?**	01 Yes 02 No 888 DK 99 NR	

Section 4: Financial Distress and Future Outlook

Now I'd like to change topics a little bit. Ask the respondent to think about their total monthly income and answer the following questions on the degree to which they felt able to make ends meet.

4.1	**Do you depend on anyone for money to meet your basis living expenses?**<br>NOTE: This means having at least one friend, partner, or relative who regularly provides financial assistance to you.	01 Yes 02 No 88 DK 99 NR			
4.2	**Please indicate if you have had any of the following financial worries in the last 6 months and whether it was mild or severe.**	01<br>NO, did not occur.	02<br>YES, occurred mildly.	03<br>YES, occurred severely.	88 DK / 99 NR
4.2A	Had trouble sleeping because of financial problems				
4.2B	Had worries about ability to make ends meet				
4.2C	Had worries about debt and having enough money				
4.2D	Had to pay a large financial bill unexpectedly				
4.2E	Concern about not affording needed health services				

4.3	**In the last 6 months, please describe how often you did NOT have enough money for each of the following living expenses?**	**Response**				
		01<br>Never	02<br>Once	03<br>2-3 Times	04<br>Many times (4+)	88/99<br>DK/NR
4.3A	Money to buy food.					
4.3B	Money to buy clothing.					
4.3C	Money for transportation.					
4.3E	Money for housing fees.					
4.3F	Money for health or medical expenses.					

	Question	Response	Skip
4.4A	**In the past 6 months, did you go to sleep at night hungry because there was not enough food?**	01 Yes 88 DK→<br>02 No→ 99 NR→	
4.4B	**How often did this happen?**	01 Once<br>02 2 or 3 times<br>03 4 or more times 88 DK NR 99	
4.5	**In the past 6 months, did you go a whole day and night without eating anything at all because there was not enough food?**	01 Yes 88 DK→<br>02 No→ 99 NR→	
4.6	**How often did this happen in the past?**	01 Once<br>02 2 or 3 times<br>03 4 or more times 88 DK 99 NR	
4.7	**In the last 6 months, have you been severely affected by ____________?**		

4.7A	Loss of employment or income	01 Yes 02 No 88 DK 99 NR	
4.7B	Loss of savings	01 Yes 02 No 88 DK 99 NR	
4.7C	Loss of housing	01 Yes 02 No 88 DK 99 NR	
4.7D	Loss of other asset used to earn money (i.e., bicycle, cell phone, car, machinery, etc.)	01 Yes 02 No 88 DK 99 NR	
4.7E	Loss of financial support from someone who had been assisting you	01 Yes 02 No 88 DK 99 NR	
Now I am going to talk about the ways you might have felt or acted over the past week. Remember that there are no right or wrong answers.			
4.8	**In the past week, please tell me whether you have felt this way rarely (less than 1 day), some (1-2 days), occasionally (3-4 days), or all of the time (5-7 days).**		
4.8A	I was bothered by things that usually don't bother me.	01 Rarely 02 Some 03 Occasionally<br>04 All of the time 88 DK 99 NR	
4.8B	I had trouble keeping my mind on what I was doing.	01 Rarely 02 Some 03 Occasionally<br>04 All of the time 88 DK 99 NR	
4.8C	I felt depressed.	01 Rarely 02 Some 03 Occasionally<br>04 All of the time 88 DK 99 NR	
4.8D	I felt that everything I did was an effort.	01 Rarely 02 Some 03 Occasionally<br>04 All of the time 88 DK 99 NR	
4.8E	I felt hopeful about the future.	01 Rarely 02 Some 03 Occasionally<br>04 All of the time 88 DK 99 NR	
4.8F	I felt fearful.	01 Rarely 02 Some 03 Occasionally<br>04 All of the time 88 DK 99 NR	
4.8G	My sleep was restless.	01 Rarely 02 Some 03 Occasionally<br>04 All of the time 88 DK 99 NR	
4.8H	I was happy.	01 Rarely 02 Some 03 Occasionally<br>04 All of the time 88 DK 99 NR	
4.8I	I felt lonely.	01 Rarely 02 Some 03 Occasionally<br>04 All of the time 88 DK 99 NR	
4.8J	I could not "get going."	01 Rarely 02 Some 03 Occasionally<br>04 All of the time 88 DK 99 NR	
4.8K	I was bothered by things that usually don't bother me.	01 Rarely 02 Some 03 Occasionally<br>04 All of the time 88 DK 99 NR	
4.8L	I had trouble keeping my mind on what I was doing.	01 Rarely 02 Some 03 Occasionally<br>04 All of the time 88 DK 99 NR	
4.8M	I felt depressed.	01 Rarely 02 Some 03 Occasionally<br>04 All of the time 88 DK 99 NR	
Now I am going to ask you a series of questions about how you are doing in general.			
4.9	**In the past week, please tell me whether you have felt this way none of the time, a little of the time, some of the time, most of the time, or all of the time?**		
4.9A	I think I am doing pretty well	01 None of the time 02 A little of the time,<br>03 Some of the time 04 Most of the time<br>05 All of the time 88 DK 99 NR	
4.9B	I can think of many ways to get the things in life that are most important to me.	01 None of the time 02 A little of the time,<br>03 Some of the time 04 Most of the time<br>05 All of the time 88 DK 99 NR	
4.9C	I am doing just as well as other people my age.	01 None of the time 02 A little of the time,<br>03 Some of the time 04 Most of the time<br>05 All of the time 88 DK 99 NR	
4.9D	When I have a problem, I can come up with lots of ways to solve it.	01 None of the time 02 A little of the time,<br>03 Some of the time 04 Most of the time<br>05 All of the time 88 DK 99 NR	

4.9E	I think the things I have done in the past will help me in the future.	01 None of the time 02 A little of the time,<br>03 Some of the time 04 Most of the time<br>05 All of the time 88 DK 99 NR	
4.9F	Even when others want to quit, I know that I can find ways to solve the problem	01 None of the time 02 A little of the time,<br>03 Some of the time 04 Most of the time<br>05 All of the time 88 DK 99 NR	
4.9G	I think I am doing pretty well	01 None of the time 02 A little of the time,<br>03 Some of the time 04 Most of the time<br>05 All of the time 88 DK 99 NR	

**Section 5: Perceived risk to HIV [O]**		
**No.**	**Question**	**Response**
5.1	**What do you think are the chances that you will get HIV in your lifetime?**	01 No chance<br>02 Some chance<br>03 Very high chance 88 DK 99 NR
5.2	**What do you think are the chances that your <u>current</u> sex partner(s) has acquired HIV?**	01 No chance<br>02 Some chance<br>03 Very high chance 88 DK 99 NR
5.3	**What do you think are the chances that your <u>past</u> sex partner(s) had acquired HIV?**	01 No chance<br>02 Some chance<br>03 Very high chance 88 DK 99 NR

Section 6: Sexual history [O]

The next set of questions are about your sex life over the last few months. As you may know, a person may get the AIDS virus through sexual activity. To help prevent the spread of AIDS, we need to know more about all the different types of sexual practices people engage in. Some of these questions need to be rather detailed and personal. Since this survey is confidential, no one will know your answers. We would appreciate your cooperation in answering these questions. If there is a question that you do not feel comfortable answering, please let me know and we can skip that question.

6.1	**Do you currently have a boyfriend/girlfriend?**	01 Yes 02 No 88 DK 99 NR	
6.2	**Have you ever had vaginal or anal sex?**<br>NOTE: This includes sex that was voluntary or involuntary.	01 Yes 88 DK→<br>02 No→ 99 NR→	
6.3	**How old were you when you first had vaginal or anal sex?**	|___||___| 88 DK 99 NR	
6.4	**Have you had vaginal or anal sex in the last 6 months?**	01 Yes 02 No 88 DK 99 NR	

The number of sexual partners people have had differs a lot from person to person. Some people report having had one sex partner, some 2 or more partners, and still others report 100s of partners.

6.5A	**In the past 6 months, how many people, including clients, have you had sexual intercourse with?**	|___||___| |___||___| 88 DK 99 NR	
6.5B	**In the past one year, how many people, including clients, have you had sexual intercourse with?**	|___||___| |___||___| 88 DK 99 NR	
6.6	**How often have you used condoms when having sex with your regular partner(s) in the last 6 months?**<br>NOTE: Regular partner(s) refers to any person you regularly have sex with.	01 No regular partner<br>02 Every time<br>03 Often<br>04 Sometimes<br>05 Rarely<br>06 Never 88 DK 99 NR	

6.7	**How often did you use condoms when you had sex with casual partners in the last month?**<br>NOTE: Casual partners means any person that you had sex with who is not a regular sexual partner and is not a paying client.	01 No casual partner<br>02 Every time<br>03 Often<br>04 Sometimes<br>05 Rarely<br>06 Never 88 DK 99 NR				
6.8	**How often have you used condoms when you have been paid for sex in the last month?**	01 No paid sex<br>02 Every time<br>03 Often<br>04 Sometimes<br>05 Rarely<br>06 Never 88 DK 99 NR				
6.9	**Was a condom used every time you had sexual intercourse in the last 6 months?**	01 Yes 02 No 88 DK 99 NR				
6.10	**During the last 6 months, have you had a disease that you got through sexual contact?**	01 Yes 02 No 88 DK 99 NR				
6.11	**Have you ever had sex while you were drunk on alcohol?**	01 Yes 02 No 88 DK 99 NR				
6.12	**Have you ever had sex while you were high on drugs?**	01 Yes 02 No 88 DK 99 NR				
6.13	**Are you (or your sexual partner) using a contraceptive method to avoid becoming pregnant?**	01 Yes 02 No 88 DK 99 NR				
6.14A	**What contraceptive method are you currently using? (Select all that apply)**	01 Pill<br>02 Injectables<br>03 Condom<br>04 Spermicides<br>05 IUD<br>06 Implants / Diaphragm<br>07 Periodic abstinence<br>08 Withdrawal<br>09 Other traditional methods<br>10 Sterilization 88 DK 99 NR				
6.14B	**If not, what are your reasons for not using a birth control method currently?**<br>**(Select all that apply)**	01 Not sexual active<br>02 Costs to purchase<br>03 Not sure where to get it<br>04 Embarassment<br>05 Concern for side effects<br>06 Partner or family disapproves<br>07 Other (specify):_______________________<br>88 DK 99 NR				
6.15	**Have you ever had sex with a stranger?**	01 Yes 02 No 88 DK 99 NR				
	**Question**	**Response**				**Skip**
6.16	**In the last 6 months, have you had sexual intercourse in exchange for these reasons?**	01<br>Yes	02<br>No	88<br>DK	99<br>NR	
6.16A	In exchange for food					
6.16B	In exchange for housing					
6.16C	In exchange for money					
6.16D	In exchange for medicines or drugs					
6.16E	In exchange for other things (specify):<br>_________________________					
6.17	**How many times have you ever exchanged sex with a person for one of these reasons? (Select one)**	01 Never<br>02 Once<br>03 2 to 3 times<br>04 4 or more times 88 DK 99 NR				
6.18A	**Within the last 2 years, have you ever been <u>offered</u> more money for sex without a condom?**	01 Yes 02 No 88 DK 99 NR				

6.18B	**Within the last 2 years, have you ever accepted more money for sex without a condom?**	01 Yes 02 No 88 DK 99 NR	
6.19A	**Within the last 2 years, have you had unprotected sex where you would have used a condom, but did not have one and did not want to wait?**	01 Yes 02 No 88 DK 99 NR	
6.19B	**On a scale of 1 to 10, with 10 being very likely and 1 being very unlikely, how likely are you to use a condom if you have to wait one day prior to getting a condom?**	1 2 3 4 5 6 7 8 9 10<br>Very unlikely to wait ... Very likely to wait	
6.19C	**On a scale of 1 to 10, with 10 being very likely and 1 being very unlikely, how likely are you to use a condom if you have to wait one week prior to getting a condom?**	1 2 3 4 5 6 7 8 9 10<br>Very unlikely to wait ... Very likely to wait	
Now, I would like for you to think about your most recent sexual partner.			
6.20	**How old is your most recent sexual partner?**	|___||___| 88 DK 99 NR	
6.21	**What is your most recent partner's gender?**	01 Male<br>02 Female<br>03 Other (specify): ____________________	
6.22	**What is your relationship to this partner? (Select one)**	01 Main partner/boyfriend/girlfriend<br>02 Regular sex partner<br>03 Non-regular/casual sex partner<br>04 Sex work client<br>05 Other (specify:) ____________________	
6.23	**Did you use a condom with your most recent partner the last time you had sex?**	01 Yes 02 No 88 DK 99 NR	
6.24	**Have you ever talked with this person about using condoms?**	01 Yes 02 No 88 DK 99 NR	
6.25	**Have you ever talked to this person about personal risk of HIV?**	01 Yes 02 No 88 DK 99 NR	
6.26	**Have you ever talked with this person about preventing HIV transmission?**	01 Yes 02 No 88 DK 99 NR	
6.27	**Have you ever asked this person to use a condom?**	01 Yes 02 No 88 DK 99 NR	
6.28	**Do you regularly receive financial support from this person?**	01 Yes 02 No 88 DK 99 NR	
6.29	**During the past 6 months, have you ever paid this person with money or material goods in exchange for sex?**	01 Yes 02 No 88 DK 99 NR	
6.30	**During the past 6 months, has this person ever paid you with money or material goods in exchange for sex?**	01 Yes 02 No 88 DK 99 NR	
6.31	**During the past 6 months, has this person had sex with anyone besides you?**	01 Yes 02 No 88 DK 99 NR	
6.32	**Has this person ever slapped you?**	01 Yes 02 No 88 DK 99 NR	
6.33	**Has this person ever hit you with their fist or with something else that could hurt you?**	01 Yes 02 No 88 DK 99 NR	
6.34	**Have you ever felt you had to have sex with this person in order to receive money, food, or housing?**	01 Yes 02 No 88 DK 99 NR	
6.35	**Have you ever felt you had to have sex with this person in order to other forms of financial support?**	01 Yes 02 No 88 DK 99 NR	
Now, I would like for you to think about your next most recent partner. We will refer to this paerson as your 2nd sex partner.			
6.36	**Have you had any other sexual partners before this most recent partner?**	01 Yes 88 DK<br>02 No 99 NR	
6.37	**How old is your second sexual partner?**	|___||___| 88 DK 99 NR	
6.38	**What is your second most recent partner's gender?**	01 Male<br>02 Female<br>03 Other (specify): ____________________	

6.39	**What is your relationship to this partner? (Select one)**	01 Main partner/boyfriend/girlfriend<br>02 Regular causal sex partner<br>03 Non-regular casual sex partner<br>04 Sex work client<br>05 Other (specify:) ____________________	
6.40	**Did you use a condom with your second most recent partner the last time you had sex?**	01 Yes 02 No 88 DK 99 NR	
6.41	**Have you ever talked with this person about using condoms?**	01 Yes 02 No 88 DK 99 NR	
6.42	**Have you ever talked to this person about personal risk of HIV?**	01 Yes 02 No 88 DK 99 NR	
6.43	**Have you ever talked with this person about preventing HIV transmission?**	01 Yes 02 No 88 DK 99 NR	
6.44	**Have you ever asked this person to use a condom?**	01 Yes 02 No 88 DK 99 NR	
6.45	**Do you regularly receive financial support from this person?**	01 Yes 02 No 88 DK 99 NR	
6.46	**During the past 6 months, have you ever paid this person with money or material goods in exchange for sex?**	01 Yes 02 No 88 DK 99 NR	
6.47	**During the past 6 months, has this person ever paid you with money or material goods in exchange for sex?**	01 Yes 02 No 88 DK 99 NR	
6.48	**During the past 6 months, has this person had sex with anyone besides you?**	01 Yes 02 No 88 DK 99 NR	
6.49	**Has this person ever slapped you?**	01 Yes 02 No 88 DK 99 NR	
6.50	**Has this person ever hit you with their fist or with something else that could hurt you?**	01 Yes 02 No 88 DK 99 NR	
6.51	**Have you ever felt you had to have sex with this person in order to receive money, food, or housing?**	01 Yes 02 No 88 DK 99 NR	
6.52	**Have you ever felt you had to have sex with this person in order to other forms of financial support?**	01 Yes 02 No 88 DK 99 NR	

Section 7: Efforts to prevent HIV [O]

The following questions of this interview are more personal and ask about your relationships and sexual history. If there is a question that you do not feel comfortable answering, please let me know and we can skip that question.

No.	Question	Response	Skip
7.1	**In your opinion, how do young adults in this settlement prevent HIV? (Select all that apply)**	01 Abstain from sex<br>02 Use condoms<br>03 Use a condom for the first time ever<br>04 Change the ways to select sexual partners<br>05 Reduce or limit the number of different sexual partners<br>06 Discuss HIV with sexual partner(s)<br>07 Discuss condoms with sexual partner(s)<br>08 Have sex with only one partner<br>09 Using PrEP (Pre-exposure prophylaxis)<br>10 Using PEP (Post-Pre-exposure prophylaxis)<br>11 Use microbicides or HIV vaccine<br>12 Voluntary medical male circumcision<br>13 Other (specify) (______________________)<br>88 DK 99 NR	

7.2	**What you have you done to prevent and/or reduce risk of HIV infection?**<br>(NOTE: DO NOT READ OPTIONS. SELECT ALL THAT ARE MENTIONED BY PARTICIPANT.)	01 Abstained from sex<br>02 Used condoms<br>03 Change the ways to select sexual partners<br>04 Reduced or limited number of sexual partners<br>05 Discuss HIV with sexual partner(s)<br>06 Discuss condoms with sexual partner(s)<br>07 Had sex with only one partner<br>08 Used PrEP (Pre-exposure prophylaxis)<br>10 Used PEP (Post-Pre-exposure prophylaxis)<br>11 Used microbicides or HIV vaccine<br>12 Underwent penile circumcision<br>13 Other (specify:) *(______________________)*<br>88 DK 99 NR				
7.3	**In the past 6 months, have you paid for condoms using your own money?**	01 Yes 02 No 88 DK 99 NR				
7.4	**About how much money have you spent on condoms in the last 6 months?**	[___|___] [___|___] [___|___] Ksh 88 DK 99 NR				
7.5	**In the past year, have you received any free condoms?**	01 Yes 02 No 88 DK 99 NR				
7.6	**Did you use the condoms that you were given?**	01 Yes 02 No 88 DK 99 NR				
	**Question**	**Response**				
7.7	**Please let me know which of the following practices you have done in the <u>last 6 months</u> because you thought it would keep you from getting or transmitting HIV?**	YES, I did and it worked.<br>01	YES, I tried, but it did <u>not</u> work.<br>02	NO, I have not tried, but it is possible.<br>03	NO, I have not tried, but and it is <u>not</u> possible.<br>04	88 DK<br>99 NR
7.7A	Used condoms every time having sex					
7.7B	Used condoms sometimes when having sex					
7.7C	Had sex with one person only					
7.7D	Had sex with one person only AND asking the person about his/her HIV status					
7.7E	Had sex with one person only who is not HIV-infected and has no other sex partner(s).					
7.7F	Obtained an HIV test for myself					
7.7G	Asked my sex partner(s) to take an HIV test					
7.7H	Discussed ways to prevent HIV with my sex partner(s)					
7.7I	Took anti-HIV medications before sex (PreP)					
7.7J	Took anti-HIV medications after sex (PeP)					
7.7K	Took anti-HIV medications each day (ART)					
7.7L	Used a topical microbicides or other pre-/post-exposure prophlyaxis					
7.7M	Changed the type of sexual partner I have					
7.7N	Reduced the number of persons I have sex with					
7.7O	Abstained from sex completely in last 6 months					
7.7P	Used microbicides					
7.7Q	Underwent medical male (penile) circumcision					
	**Question**	**Response**				**Skip**
7.8	In your opinion, are financial incentives likely to motivate greater adoption of efforts to prevent HIV in young adults living in your settlement?	01 Yes 02 No 88 DK 99 NR				
7.9	What amount of conditional cash incentives each month would be very effective?	|___||___| |___||___|ksh 88 DK 99 NR				

7.10	What amount of conditional cash incentives would be somewhat effective?	|___||___| |___||___|ksh 88 DK 99 NR	
7.11	What amount of conditional cash incentives would be only a little effective?	|___||___| |___||___|ksh 88 DK 99 NR	

Section 8: HIV-related careseeking [O]
The following questions of this interview are more personal and ask about your relationships and sexual history. If there is a question that you do not feel comfortable answering, please let me know and we can skip that question.

No.	Question	Response	Skip
8.1	**Have you ever been tested for the HIV virus?**	01 Yes 02 No 88 DK 99 NR	
8.2	**How many months ago was your last HIV test?**	[___|___] Months 88 DK 99 NR	
8.3	**Was that the first time you had ever been tested?**	01 Yes 02 No 88 DK 99 NR	
8.4	**If your lifetime, how many times have you been tested for HIV?**	[___|___] Number of times 88 DK 99 NR	
8.5	**When you were last tested for HIV, did you share your results with anyone?**	01 Yes 02 No 88 DK 99 NR	
8.6	**Whether negative or positive, who have you shared your test results with? (Select all that apply)**	01 Sexual partner<br>02 Friend (non-sexual partner)<br>03 Relative<br>04 Employer<br>05 Health Professional<br>06 Other (specify): ____________________	
8.7	**Where did you receive your last HIV test?**<br>NOTE: Clinical settings include private doctor's office, hospital, public health clinic or community health center. Nonclinical settings include HIV counseling and testing site, street outreach program or mobile unit, or home.	01 Clinical setting<br>02 Nonclinical setting<br><br>88 DK 99 NR	
8.8	**How much money did you spend for each of the following?**		
8.8A	Fee for HIV test service	[___|___] [___|___] [___|___] Ksh 88 DK 99 NR	
8.8B	Other clinic fees related to HIV test	[___|___] [___|___] [___|___] Ksh 88 DK 99 NR	
8.8C	Transport fare to testing site	[___|___] [___|___] [___|___] Ksh 88 DK 99 NR	
8.8D	Food while traveling to test site	[___|___] [___|___] [___|___] Ksh 88 DK 99 NR	
8.8E	Pay for child care	[___|___] [___|___] [___|___] Ksh 88 DK 99 NR	
8.8F	Other costs (specify):________________________	[___|___] [___|___] [___|___] Ksh 88 DK 99 NR	
8.9	**About how many hours did you spend from start to finish to obtain your HIV? (i.e., including travel and waiting time)**	[___|___] Hours 88 DK 99 NR	
8.10	**How about how much money would you have earned in the time it took you to test for HIV?**	[___|___] [___|___] [___|___] Ksh 88 DK 99 NR	
8.11	**In the next 3 months, do you intend to use any of the following services for concerns related to HIV/AIDS?** (Select all that apply)	01 AIDS information services<br>02 HIV/AIDS counseling<br>03 HIV testing<br>04 HIV/AIDS support group<br>05 Folk healers, traditional medicine<br>06 Medical services other than HIV testing<br>07 Public assistance<br>08 Legal services<br>09 Other (specify): _______________88 DK 99 NR	

8.12	**In the last 12 months, have you had a one-on-one conversation with an outreach worker or counselor about ways to prevent HIV?**<br>NOTE: This excludes conversations that were part of HIV testing.	01 Yes 02 No 88 DK 99 NR	
8.13	**In the last 12 months, have you participated in a small-group discussion that is part of an organized session about ways to prevent HIV?**<br>NOTE: This excludes informal discussions with friends.	01 Yes 02 No 88 DK 99 NR	
8.14	**Have you heard of an oral HIV self-test?**<br>NOTE: This is an HIV test a person can do themselves at home using their saliva and receive the results in 20 minutes.	01 Yes 02 No 88 DK 99 NR	
8.15	**Would you be willing to use an HIV self-test in the future?**	01 Yes 02 No 88 DK 99 NR	
8.16	**If <u>YES</u>, what would be some possible reasons to use the HIV self-test?**<br>**(Select all that apply)**	01 Knowledge of HIV status<br>02 Increased confidentiality<br>03 Increased convenience<br>04 Autonomy, ability to do it one self<br>05 Less stigma<br>06 Less risk of status disclosure<br>07 Saves time<br>08 Saves money<br>09 Easier to repeat testing<br>10 Accessed through more outlets<br>11 Other (specify): ______________________<br>88 DK 99 NR	
8.17	**If <u>NO</u>, what would be some possible reasons to not use the HIV self-test?**<br>**(Select all that apply)**	01 Too costly, expensive<br>02 Possible inaccurate results<br>03 Lack of counseing<br>04 Difficulty with referral to care, treatment<br>05 Facility HIV testing is free<br>06 Unsafe disposal<br>07 Possible self-harm<br>08 Used with coercion<br>09 Ill-omened use of money<br>10 Encourages unprotected sex<br>11 Other (specify): _____________________<br>88 DK 99 NR	
8.18	**Would you be comfortable asking your sexual partner(s) to use a self-test before you had sex?**	01 Yes 02 No 88 DK 99 NR	
8.19	**Where would you prefer to go and collect an HIV self-test kit?**<br>**(Select one)**	01 HIV testing clinic<br>02 Hospital<br>03 Pharmacy or laboratory<br>04 Community center<br>05 School of university<br>06 Work place<br>07 Other (specify): ______________ 88 DK 99 NR	
8.20	**Do you currently have access to cell phone?**	01 Yes 02 No 88 DK 99 NR	
8.21	**Do you regularly share your cell phone with anyone else?**	01 Yes 02 No 88 DK 99 NR	
8.22	**After taking an HIV self-test, would you be willing to receive HIV counseling via your cell phone?**	01 Yes 02 No 88 DK 99 NR	
8.23	**Can you access the internet via your cell phone?**	01 Yes 02 No 88 DK 99 NR	
8.24	**Have you ever received text messages on your cell phone?**	01 Yes 02 No 88 DK 99 NR	
8.25	**Have you ever received text messages related to HIV prevention?**	01 Yes 02 No 88 DK 99 NR	

8.26	**Have you ever received text messages related to HIV care or treatment?**	01 Yes 02 No 88 DK 99 NR	
8.27	**Have you ever received text messages related to improving your financial status?**	01 Yes 02 No 88 DK 99 NR	
8.28	**In a given month, how much do you spend to keep your phone charged and with air time?**	[___|___] [___|___] [___|___] Ksh 88 DK 99 NR	

**Section 9: Behavioral economics**		
9.1	**Imagine you won a prize and had to choose between receiving the 50,000 Ksh ($500 USD) tomorrow, or 75,000 Ksh ($750 USD) in one month. Which would you choose?** (Select one)	01 50,000 KSH tomorrow<br>02 75,000 KSH in one month<br>88 DK 99 NR
9.2	**What if instead 100,000 Ksh ($1,000 USD) were offered one month from now? What would you prefer?** (Select one)	01 50,000 KSH tomorrow<br>02 100,000 KSH in one month<br>88 DK 99 NR
9.3	**What if instead 60,000 Ksh ($600 USD) were offered tomorrow? What would you prefer?** (Select one)	01 60,000 KSH tomorrow<br>02 75,000 KSH in one month<br>88 DK 99 NR
9.4	**Okay, tell me this. Suppose you had just won a prize. Would you choose to receive 500 Ksh ($5 USD) tomorrow or 1,000 Ksh ($10 USD) in one year?** (Select one)	01 550 KSH tomorrow<br>02 1,000 KSH in one year<br>88 DK 99 NR
9.5	**If you have had 10,000 Ksh ($100 USD) today, how would you spend it?** (Select one)	01 Spend all of it<br>02 Spend most of it<br>03 Spend half, save half<br>04 Save most of it<br>05 Save all of it 88 DK 99 NR
9.6	**Do you know someone personally who is living with HIV?**	01 Yes 02 No 88 DK 99 NR
9.7	**Do you know anyone who has benefited from antiretroviral (ARV) medications to treat HIV?**	01 Yes 02 No 88 DK 99 NR
9.8	**Did you hear about this benefit within the last year or over a year ago?**	01 Within the last year<br>02 Over a year ago 88 DK 99 NR
9.9	**Do you know anyone who has benefited from learning about his/her HIV status through voluntary counseling and testing?**	01 Yes 02 No 88 DK 99 NR
9.10	**Did you hear about this benefit within in the last year or over a year ago?**	01 Within the last year<br>02 Over a year ago 88 DK 99 NR
9.11	**Do you have a close friend or family member who has died from AIDS?**	01 Yes 02 No 88 DK 99 NR
9.12	**Did the person die within in the last year or over a year ago?**	01 Within the last year<br>02 Over a year ago 88 DK 99 NR
9.13	**Do you know of anyone in your household who has previously tested for HIV?**	01 Yes 02 No 88 DK 99 NR
9.14	**To your knowledge, was their most recent HIV test within the last year or over a year ago?**	01 Within the last year<br>02 Over a year ago 88 DK 99 NR
	**Question**	**Response**
9.15	**How do you see yourself: Please use a scale from 0 to 10, where 0 means "does not describe me at all" and a 10 means "describes me perfectly"**	
9.15A	**I generally tend to take risks**	Doesn't describe me at all Describes me perfectly<br>0 1 2 3 4 5 6 7 8 9 10
9.15B	**I tend to postpone things even though it would be better to get them done right away.**	Doesn't describe me at all Describes me perfectly<br>0 1 2 3 4 5 6 7 8 9 10

9.15C	**I go without some things today so that I will be able to afford more tomorrow.**	Doesn't describe me at all Describes me perfectly<br>0 1 2 3 4 5 6 7 8 9 10
9.16	**Imagine you can win a prize at a game. Would you prefer to get:** (Select one)	01 5,000 KSh for sure<br>02 Have a chance of 1 in 10 to win 40,000 KSh<br>03 Have a chance of 1 in 100 to win 300,000 Ksh<br>88 DK 99 NR
9.17	**Imagine you made a bet and you lost. Would you rather lose:** (Select one)	01 5,000 KSh for sure<br>02 Have a chance of 1 in 10 to lose 40,000 KSh<br>03 Have a chance of 1 in 100 to lose 300,000 Ksh<br>88 DK 99 NR

Now I am going to talk about the ways your personal finances influence your sexual behaviors. Remember that there are no right or wrong answers.

9.18	**In the last 12 months, please let me know if the following scenario has happened to you. You can respond by saying true or false.**	
9.18A	I use extra money that I receive to acquire more sex partners.	01 True 00 False 88 DK 99 NR
9.18B	The number of sex partners I have decreses when my money is low.	01 True 00 False 88 DK 99 NR
9.18C	If I were HIV-positive, I feel confident that I would have sufficient money to pay for my treatment costs.	01 True 00 False 88 DK 99 NR
9.18D	I am not concerned about getting HIV because if I were infected, I could still live a healthy life.	01 True 00 False 88 DK 99 NR
9.18E	I would agree to unprotected sex if the person was willing to pay me.	01 True 00 False 88 DK 99 NR
9.18F	Male condoms are expensive.	01 True 00 False 88 DK 99 NR
9.18G	HIV testing is expensive.	01 True 00 False 88 DK 99 NR
9.18H	Female condoms are expensive.	01 True 00 False 88 DK 99 NR
9.18I	I cannot think about avoiding HIV because I have more important financial worries.	01 True 00 False 88 DK 99 NR
9.18J	I have a sugar-mommy or sugar-daddy.	01 True 00 False 88 DK 99 NR
9.18K	I am a sugar-mommy or sugar-daddy.	01 True 00 False 88 DK 99 NR

Section 10. Household socioeconomic status

We are now at the last set of questions. I am going to ask you about the finances of your household. This refers to the sum of all people who are currently living with you. If there is a question that you do not feel comfortable answering, please let me know and we can skip that question.

No.	Question	Response	Skip
10.1A	**Does your household (family) have any cash savings?**	01 Yes 02 No 88 DK 99 NR	
10.1B	**If yes, approximately how much savings?**	|___||___| |___||___| ksh 88 DK 99 NR	
10.2A	**Over the past 12 months, did your household (family) borrow cash from an institution?**	01 Yes 02 No 88 DK 99 NR	
10.2B	**If yes, how much was borrowed?**	|___||___| |___||___| ksh 88 DK 99 NR	
10.2C	**What did your household (family) use this borrowed money for?**	01 Start a business<br>02 Household expenses<br>03 Medical expenses<br>04 Education expenses<br>05 Other (specify): _______________<br>88 DK 99 NR	
10.3A	**Does any member of your household have money that they borrowed from an individual and will have to repay?**	01 Yes 02 No 88 DK 99 NR	
10.3B	**If yes, approximately how much debt do they owe that person(s)?**	|___||___| |___||___|ksh 88 DK 99 NR	

**Section 10. Household socioeconomic status**<br>*We are now at the last set of questions. I am going to ask you about the finances of your household. This refers to the sum of all people who are currently living with you. If there is a question that you do not feel comfortable answering, please let me know and we can skip that question.*						
**No.**	**Question**	**Response**				**Skip**
10.3C	**Have you borrowed money from others to meet your own or family members' medical and health care expenses?**	01 Yes 02 No 88 DK 99 NR				
10.4	**In the last month, did your household (family) receive any assistance in kind (i.e., food, clothing, other items) from persons who are not members of your household?**	01 Yes 02 No 88 DK 99 NR				
10.5	**Did your family lend money to others? If yes, how much?**	01 Yes 02 No 88 DK 99 NR				
10.6	**What was the cash earned by your entire household (family) in the last month?**	KSH 25,000 OR LESS……………01<br>KSH 25,001 – 50,000……………..02<br>KSH 50,001 – 75,000……………..03<br>KSH 75,001 – 100,000……………04<br>KSH 100,001 – 150,000………....05<br>KSH 150.001 – 200,000…………..06<br>KSH 200,001 – 300,000…………07<br>KSH 300,001 – 400,000…………..08<br>KSH 400,001 – 500,000…………..09<br>KSH 500,001 OR MORE…………10<br>88 DK 99 NR				
10.7	**Who owns the house you live in?**<br>**(Select one)**	01 Self-owned<br>02 Owned by parent/guardian<br>03 Owned by other relative or friend<br>04 Rented (not owned)<br>05 Not rented and not owned<br>06 Other (specify): ________________				
10.8	**Thinking of your household's total monthly income, how easily would you say that your household is able to make ends meet?**<br>**(Select one)**	01 Very easily<br>02 Somewhat easily<br>03 Neither easily or difficultly<br>04 With some difficulty<br>05 With great difficulty<br>88 DK 99 NR				
10.9	**And to finish off this part of our conversation, do you think that one year from now your family will live better than today or worse?**	01 Will live better 88 DK<br>02 No change 99 NR<br>03 Will live worse				
10.10	**Imagine a 9-step ladder where on the bottom, the first step, stand the poorest people, and on the highest step, the 9th, stand the rich. On which step is your family located today?**	Poorest Richest<br>1 2 3 4 5 6 7 8 9				
		01-Yes	02-No	88-DK	99-NR	Skip
10.11	**Does your household have any of the following?**					
10.11A	Tap drinking water					
10.11B	Electricity					
10.11C	Television					
10.11D	Electric/gas cooking stove					
10.11E	Radio					
10.11F	Cellular phone					

ID: __________-___
Date: ___/____/_____

Section 10. Household socioeconomic status<br>*We are now at the last set of questions. I am going to ask you about the finances of your household. This refers to the sum of all people who are currently living with you. If there is a question that you do not feel comfortable answering, please let me know and we can skip that question.*			
**No.**	**Question**	**Response**	**Skip**
10.12A	**Thank you for your time in answering this survey. My last question relates to your safety as a result of participating in this study.**<br><br>**Did you experience any past threats to your safety as a result of participating in this study?**	01 – Yes 02 – No 88 DK 99 NR	
10.12B	**Do you expect that you will experience any future threats to your safety as a result of participating in this study?**	01 – Yes 02 – No 88 DK 99 NR	

THIS IS THE END OF THE INTERVIEW
Thank you for taking the time to talk with me today.
Do you have any additional comments or questions you would like to ask?

TO BE COMPLETED BY INTERVIEWER ONLY:

11.1	Please note the completion status of this interview	01 – Completed<br>02 – Not Completed
11.2	What were the reasons for non-completion?	01 – Not enough time<br>02 – Interruption(s)<br>03 – Participant was distressed<br>04 - Participant was fatigued<br>05 – Participant requested to end survey<br>88 DK 99 NR
11.3	Did the participant agree to recruit additional peers?	
11.4	What reasons were provided in declining to recruit other peers?	01 – Not comfortable<br>02 – Does not know any one<br>03 – Will be away or busy<br>04 – Other (specify): ________________________<br>88 DK 99 NR
11.5	How many recruitment coupons were provided?	|__|__| number of coupons given to participant
11.6	How willing is the participant to distribute coupons to their peers?	Not Willing Very Willing<br>1 2 3 4 5
11.7	Please fill-in the recruitment coupon codes:	|__|__| |__|__| 1st coupon<br>|__|__| |__|__| 2nd coupon<br>|__|__| |__|__| 3rd coupon<br>|__|__| |__|__| 4th coupon
