# Supplementary material for: Lessons learned from using respondent-driven sampling (RDS) to assess sexual risk behaviors among Kenyan young adults living in urban slum settlements: A process evaluation
Source: PLoS One. 2020 Apr 10;15(4):e0231248. doi: 10.1371/journal.pone.0231248 (PMC7147752; doi:10.1371/journal.pone.0231248)
Supplement: S2 Questionnaire — (PDF) [file pone.0231248.s002.pdf]

ID: __________-___
Tarehe: ___/____/_____

KICHWA CHA UTAFITI
Muktadha wa kiuchumi na hatari za kuambukizwa virusi vinayosababisha ukimwi kwa vijana wanoishi katika makazi duni mijini humu Kenya
DODOSO YA UTAFITI
Vijana wa Miaka ya 18 to 22

**KAMILISHA KABLA NA BADAYA YA MAHOJIANO :**	
**01 KITAMBULISHO CHA MSHIRIKI**	|___|___|___|___|
**02 KITAMBULISHO CHA MHOJI**	|___|___|
**03 TAREHE YA KUHOJIWA**	_____/______/_______<br>Siku Mwezi Mwaka
**04 SIKU YA KUHOJIWA**	1 – Jumatatu 2 – Jumanne 3 – Jumatano 4 – Alhamisi 5 – Ijumaa 6 – Jumamosi<br>7 – Jumapili
**05 SAA YA KUANZA**	_____:_____<br>Saa Dakika
**06 SAA YA KUMALIZA**	_____:_____<br>Saa Dakika
**07 MUDA WA MAHOJIANO**	_______ chini au sawa na dakika 90 _______ kupita dakika 90
**08 MTAA**	1 = Korogocho 2 = Kawangware (Select one)
**09 LUGHA**	1 = English 2 = Kiswahili (Select one)

Section 1:Sifa za Demographia
Kabla tuanze, ningependelea kukuelezea vile mahojiano haya yatafaniyka. Vilivyo elezewa mwanzoni, ushirikiano wako na kila kipengele cha utafiti huu ni kwa hiari. Unaweza wacha/ruka swali usilotaka kujibu, lakini tunafahamu kwakujitolea.Unaweza uliza swali lolote kufafanua ambapo hujaelewa au unaweza kukomesha mahojiano haya wakati wowote.Kumalizia, taarifa yoyote unayopata hapa kwa utafiti huu, ni ya kisiri. Majibu yako yatajulikana kwa nambari na sio jina lako. Ningependela kuanza na maswali ya kawaida.

**No.**	**Swali**	**Jibu**	**Ruka**
1.0A	**Unauhusiano gani na aliyekupa dodoso hii? (Chagua jibu moja)**	01 Rafiki<br>02 Familia<br>03 Jirani<br>04 Hatuna Uhusiano<br>05 Nyengineo (taja):_________________<br>88 DK 99 NR 77 Not-Applicable (NA)	
1.0B	**Umemjua huyu mtu kwa muda gani?**	Miaka |___||___| 88 DK 99 NR 77NA	
1.1	**Jinsia**	01 Mwanamume<br>02 Mwanamke<br>03Nyinginezo (taja): ___________________	
1.2	**Una miaka mingapi?**	Miaka |___||___| 88 DK99 NR	
1.3	**Ume soma hadi kiwango kipi?**	00 Sijawahi enda shuleni→<br>01 Shule ya Msingi<br>02 Shule ya Sekondari<br>03 Elimu ya Juu 88 DK99 NR	
1.4	**Kwa sasa hivi umejiunga na shule yeyote?**	01 Ndio 02 La 88 DK 99 NR	
1.5	**Umeishi katika makazi haya kwa muda gani?**	Miaka [___|___] Miezi [___|___]<br>88 DK 99 NR	
1.6	**Ume hama nyumba yako marangapi katika mwaka huu?**<br>NOTE: Aidha ndani au nje ya Makazi haya.	[___|___] mara ngapi<br>88 DK 99 NR	

ID: __________-___
Tarehe: ___/____/_____

**Section 1:Sifa za Demographia**<br>*Kabla tuanze, ningependelea kukuelezea vile mahojiano haya yatafaniyka. Vilivyo elezewa mwanzoni, ushirikiano wako na kila kipengele cha utafiti huu ni kwa hiari. Unaweza wacha/ruka swali usilotaka kujibu, lakini tunafahamu kwakujitolea.Unaweza uliza swali lolote kufafanua ambapo hujaelewa au unaweza kukomesha mahojiano haya wakati wowote.Kumalizia, taarifa yoyote unayopata hapa kwa utafiti huu, ni ya kisiri. Majibu yako yatajulikana kwa nambari na sio jina lako. Ningependela kuanza na maswali ya kawaida.*			
**No.**	**Swali**	**Jibu**	**Ruka**
1.7	**Hali yako ya Ndoa ?**	01 Olewa or Kuishi pamoja bila ndoa<br>02 Mjane<br>03 Talaka/Tengamana<br>04 kutoolewa88 DK 99 NR	
1.8	**Kabila lako ni lipi?**	01 DhoLuo<br>02 MLuhya<br>03 MKikuyu<br>04Nyinginezo (taja): __________________	
1.9	**Dini yako ni ipi?**	01 Mkristo<br>02 Muislamu<br>03 Sijihusishi na dini<br>04 Nyinginezo (taja):___________________	
1.10	**Watu wangapi wanaishi kataika nyumba yako? (Pamoja na wewe mwenyewe)**	|___||___| Idadi ya watu<br>88 DK 99 NR	

**Section 2: Mapato ya Kikazi**<br>*Hauta ulizwa kuhusu mapato yako adha kutokana kujiajiri au kuajiriwa na mtu yeyote mwingine*			
**No.**	**Swali**	**Jibu**	**Ruka**
2.1	**Katika miezi 6iliiyopita,uliwahi fanya shughuli yoyote kwa ajili ya kulipwa, faida au biashara yako badilishana?**	01 Ndio 02 La 88 DK 99 NR	
2.2	**Katika siku 7 zilizopita,uliwahi fanya shughuli yoyote kwa ajili ya kulipwa, faida au biashara yako badilishana?**	01 Ndio 02 La 88 DK 99 NR	
2.3	**Ni wiki au miezi mingapi tangu kufanya shughuli yoyote kwa ajili ya kulipwa, faida au biashara yako badilishana?**	wiki [___|___]Miezi [___|___]<br>88 DK 99 NR	
2.4	**Ni masaa mangapi kwa kila siku unayo tumia kikazi kwa kutengeneza hela au vifaa vya kujihumudu?**	[___|___] Masaa<br>88 DK 99 NR	
2.5	**Ni masaa mangapi kila jumamosi au jumapili unayo tumia kikazi kwa kutengeza hela au vifaa kujihumudu?**	[___|___] Masaa<br>88 DK 99 NR	
2.6	**Mwaka uliopita kuna biashara yoyote uliyoendeleza au kufanya shughuli yoyote ya kujiajiri?**	01 Ndio 02 La 88 DK 99 NR	
2.7	**Ni biashara ipi unayoendeleza? (Chagua jibu iliyofaa)**	01 Udreva, Usafiri<br>02 Uuzajiwa vifaa<br>03 Hudumu (Vyakula au Nguo)<br>04Burudani<br>05Huduma za nyumbani<br>06Nyinginezo(Taja):____________________	

**Section 2: Mapato ya Kikazi**<br>*Hauta ulizwa kuhusu mapato yako adha kutokana kujiajiri au kuajiriwa na mtu yeyote mwingine*			
**No.**	**Swali**	**Jibu**	**Ruka**
2.8	**Ulipata faida ya pesa ngapi kutokana na biashara unayo endeleza kwa mwezi mmoja ?**<br>(Chagua jibu moja)	KSH 25,000 au chini…..…………01<br>KSH 25,001 – 50,000……………..02<br>KSH 50,001 – 75,000……………..03<br>KSH 75,001 – 100,000……………04<br>KSH 100,001 – 150,000………....05<br>KSH 150.001 – 200,000…………..06<br>KSH 200,001 – 300,000……….….07<br>KSH 300,001 – 400,000…………..08<br>KSH 400,001 – 500,000…………..09<br>KSH 500,001 na kuzidi……………10<br>88 DK 99 NR	
2.9	**Umefanya kazi yoyote ya kibarua katika mwaka uliopita?**<br>NOTE: Malipo haya yanaweza kuwa ya kifedha au aina nyingine.	01 Ndio 02 La→ 88 DK→ 99 NR→	
2.10	**Umefanya aina gani ya kibarua katika mwaka uliopita?**<br>NOTE: Chagua jibu inayofaa kibarua uliyoifanya kwa mda mrefu kuliko zingine.	01 Udreva, Usafiri<br>02 Uuzaji wa vifaa<br>03 Mhudumu (Vyakula au Nguo)<br>04 Burudani<br>05 Huduma za nyumbani<br>06 Nyinginezo(Taja):____________________	
2.9	**Ulipata kulipwa pesa ngapi kwa kijumla kwa vibarua vyote ulivyovifanya kwa mwezi moja mwaka uliopita?**<br>**(**Chagua jibu moja**)**	KSH 25,000 au chini……….……01<br>KSH 25,001 – 50,000……………..02<br>KSH 50,001 – 75,000……………..03<br>KSH 75,001 – 100,000……………04<br>KSH 100,001 – 150,000………....05<br>KSH 150.001 – 200,000…………..06<br>KSH 200,001 – 300,000……….….07<br>KSH 300,001 – 400,000…………..08<br>KSH 400,001 – 500,000…………..09<br>KSH 500,001 na kuzidi……………10<br>88 DK 99 NR	
2.10	**Unaweza kusema umeajiriwa kwa sasa?**	01 Ndio 02 La→ 88 DK→ 99 NR→	
2.11	**Aina gani za kijira zifwatazo zinaeleza hali yako ya kiajira ya sasa?**<br>**(**Chagua Moja**)**	01 Ajira ya Kudumu<br>02 Kibarua<br>03Kujiajiri (jua kali)<br>04 Kutokowa na Ajira<br>05 Nyinginezo (Taja):____________________<br>88 DK 99 NR	
2.12	**Katika kipindi cha miezi sita iliyopita,ulipata fedha zako kivipi au wapi?**<br>**(**Chagua Moja**)**	01 Biashara ya kibinafsi<br>02 Vibarua (malipo kwa kazi)<br>03 Hiba (msaada wa watoto, msaada wa walemavu)<br>04Zawadi kutoka kwa mpenzi wa ngono<br>05 Zawadi kutoka kwa familia au marafiki<br>06 Biashara ya ngono<br>07 Hukuwa na pesa yeyote<br>08Nyinginezo (Taja):<br>_____________________<br>88 DK 99 NR	
2.13A	**Mapato yako ya kila mwezi ni kiasi gani?**	|___||___| |___||___|ksh 88 DK 99 NR	

Section 2: Mapato ya Kikazi<br>*Hauta ulizwa kuhusu mapato yako adha kutokana kujiajiri au kuajiriwa na mtu yeyote mwingine*			
**No.**	**Swali**	**Jibu**	**Ruka**
2.13B	**Ungependela mapato yako ya kila mwezi kuwa kiasi gani?**	|___||___| |___||___|ksh 88 DK 99 NR	
2.14	**Ni nani ambaye ana amua vile fedha zako zitatumika?**<br>(Chagua moja)	01 Mhojiwa<br>02 Pamoja na Mpezi wa ngono<br>03 Mpenzi wa Ngono pekeyake<br>04 Pamoja na mtu mwingine<br>05 mtu mwingine pekeyake<br>88 DK 99 NR	
2.15	**Katikia mwezi mmoja, familia na marafiki hukupatia fedha kiasi gani?**	|___||___| |___||___|ksh 88 DK 99 NR	
2.16	**Katika kipindi cha miezi sita iliyopita mapato yako yamebadilika kivipi? (i.e.kuongezeka, kupunguka au kutobadilika)**	01 Ongeeka 03 Kutobadilika→<br>02 Punguka 99 NR→	
2.17	**Kutoka mwezi uliopita, mapato yako yalibadilika kiasi gani?**	|___||___| |___||___| ksh 88 DK 99 NR	
2.18	**Kwa sasa uko na akaunti ya benki?**	01 Ndio 02 La 88 DK 99 NR	

Section 3: Akiba na Madeni			
3.1	**Wewe ni mwanachama wa kikundi cha akiba?**	01 Ndio 02 La 88 DK 99 NR	
3.2	**Katika kipindi cha miezi kumi na miwili iliyopita , umewahi kuweka akiba za fedha au mali.**<br>NOTE: Hii ni pamoja na fedha au mali katika benki au mahali pengine ya kibinafsi.	01 Ndio 02 La 88 DK 99 NR	
3.3	**Katiki kipindi cha miezi sita iliyopita, umeweka kando fedha za kuweka akiba?**<br>NOTE: Hii ni pamoja na fedha au mali katika benki au mahali pengine ya kibinafsi .	01 Ndio 02 La 88 DK 99 NR	
3.4	**Ni jumla ya fedha kiasi gani umeweka akiba kwa sasa? (Jibu kwa shilling ya Kenya)**	|___||___| |___||___| ksh 88 DK 99 NR	
3.5	**Una fedha zozote umekopa na kutakikana kulipa mtu au kundi lolote?**	01 Ndio 02 La 88 DK 99 NR	
3.6	**Una deni ya kiasi gani?**	|___||___| |___||___| ksh 88 DK 99 NR	
3.7	**Una daiwa fedha kiasi gani?**	|___||___| |___||___| ksh 88 DK 99 NR	
3.8	**Katika kipindi cha miezi kumi na miwili ilyopita, umekopa fedha kutoka taasisi yoyote?**	01 Ndio 02 La→ 88 DK 99 NR	
3.9	**Ulikopa fedha kiasi gani?**	|___||___| |___||___|___||___|ksh<br>88 DK 99 NR	
3.10	**Ulitumia fedha ulizozikopa kivipi?**	01 Kuanzisha biashara<br>02 Utumizi wa nyumbani<br>03 Gharama za matibabu<br>04 Gharama za elimu<br>05 Nyenginezo (taja):____________<br>88 DK 99 NR	
3.11	**Katika kipindi cha miezi kumi na miwili ilyopita, umekopa fedha kwa familia, mpenzi au rafiki?**	01 Ndio 02 La 88 DK 99 NR	

3.12	**Ni jumla ya fehda kiasi gani unatakikana kulipa au kusawazisha madeni yako?**	|___||___| |___||___| ___||___|ksh<br>88 DK 99 NR	
3.13	**Ukihitaji mkopo wa sillingi elfu tano(dollar hamsini) leo, ungeweza kutekeleza ombi hilo?**	01 Ndio 02 La 888 DK 99 NR	
3.14	**Ukihitaji mkopo wa sillingi elfu kumi(dollar mia moja) leo, ungeweza kutekeleza ombi hilo?**	01 Ndio 02 La 888 DK 99 NR	

Section 4: Dhiki au Shida ya Fedha

Ningependa kubadilisha mwelekeo kidogo: Uliza mwenye kujibu a fikirie jumla ya mapato ya kila mwezi na kujibu maswali yafuatayo kwa kiwango walijipata wanaweza jikidhi kwa maisha yao.

4.1	**Unategemea mtu yeyote kwa fedha za kujikidhi kwa maisha ya kila siku ?**<br>NOTE: Kumainisha mtu unayetegema mara kwa mara kifedha, mpenzi, rafiki au familia.	01 Ndio 02 La 88 DK 99 NR			
4.2	**Tafadhali onyesha ikiwa umekuwa na wasi wasi wa kifedha katika miezi 6 na kuwa ilikuwa kali au kiwango ya chini.**	01<br>La, haikutokea.	02<br>Ndio, Ilitokea kidogo.	03<br>Ndio, Ilitokea kiwango ya juu.	88 DK / 99 NR
4.2A	Umepata shida ya kulala kwasababu ya shida za fedha?				
4.2B	Umepata wasiwasi kuhusu uwezo wakujikidhi kwa mahitaji ya kila siku				
4.2C	Umepata wasiwasi juu ya madeni na kuwa na fedha yakutosha				
4.2D	Umelazimika kulipa bili kubwa ya kifedha bila kutarajia				
4.2E	Wasiwasi kuhusu kupata huduma za afia zinazohitajika				

4.3	**Katika kipidi cha miezi 6 iliyopita, tafadhali eleza jinsi haukuwa na fedha za kutosheleza gharama zifuatazo?**	**Jibu**				
		01<br>Kamwe	02<br>Mara moja	03<br>Mara 2-3	04<br>Mara Nyingi (4+)	88/99<br>DK/NR
4.3A	Fedha za kununu chakula.					
4.3B	Fedha za kununua nguo.					
4.3C	Fedha za usafiri wa kila siku.					
4.3E	Fedha za kulipa kodi.					
4.3F	Fedha za kupata huduma za afia na kulipia matibabu.					

	**Swali**	**Jibu**	**Ruka**
4.4A	**Katika kipidi cha miezi 6 iliyopita, uliwai kulala nja, kwasababu ya kukosa chakula cha kutosha?**	01 Ndio 88 DK→<br>02 La→ 99 NR→	
4.4B	**Ulilala nja mara ngapi?**	01 Mara Moja<br>02 Mara 2 or 3<br>03 Mara 4 na kuzidi 88 DK NR 99	
4.5	**Katika kipidi cha miezi 6 iliyopita ,uliwahi kaa njaa mchana na siku nzima kwasababu ya kutokuwa na chakula cha kutosha?**	01 Ndio 88 DK→<br>02 La → 99 NR→	
4.6	**Ilifanyika mara ngapi?**	01 Mara Moja<br>02 Mara 2 or 3<br>03 Mara 4 na kuzidi 88 DK NR 99	
4.7	**Katika kipidi cha miezi 6 iliyopita ,umeathirika kwa kiasi cha juu cha zifuatazo ____________?**		
4.7A	Kupoteza ajira au mapato kutokana na ajira	01 Ndio 02 La 88 DK 99 NR	
4.7B	Kupoteza akiba	01 Ndio 02 La 88 DK 99 NR	
4.7C	Kupoteza makao	01 Ndio 02 La 88 DK 99 NR	
4.7D	Kupoteza mali unavyo vitumia kupata fedha au	01 Ndio 02 La 88 DK 99 NR	

	mapato (i.e., baiskeli, simu ya rununu, gari, mashine, etc.)		
4.7E	Kupoteza msaada wa kifedha kutoka kwa mtu amabaye amekuwa ana kusaidia.	01 Ndio 02 La 88 DK 99 NR	

**Section 5: Utambulizi wa Hatari kwa Virusi Vinavyosababisha Ukimwi(HIV)[O]**		
**No.**	**Swali**	**Jibu**
5.1	**Unafikiria yakuwa kuna uwezekano utawahi ambukizwavirusi vinavyosababisha ukimwi maishani mwako?**	01 Hakuna uwezekano<br>02 Kuna uwezekano<br>03 Uwezekano wa Juu sana 88 DK 99 NR
5.2	**Unafikiria yakuwa kuna uwezekano yakuwa m/wapenzi wako wa sasa wa ngono wame au ameambukizwa virusi vinavyosababisha ukimwi?**	01 Hakuna uwezekano<br>02 Kuna uwezekano<br>03Uwezekano wa Juu sana 88 DK 99 NR
5.3	**Unafikiria yakuwa kuna uwezekano yakuwa m/wapenzi wako wa kitambo wa ngono w/ameambukizwa virusi vinavyosababisha ukimwi?**	01 Hakuna uwezekano<br>02 Kuna uwezekano<br>03Uwezekano wa Juu sana 88 DK 99 NR

**Section 6: Historia ya Kimapenzi[O]**<br>*Maswali yafuatayo yanahusiana na maisha yako ya kimapenzi. Unavyo jua,mtu yeyote anaweza ambukizwa virusi vya ukumwi kupitia kitendo cha ngono.Kuzuia kuenea kwa ukimwi, tunawajibika kujua tabia tofauti za watu kuhusiana na tabia na mazoea ya ngono. Baadhi ya maswali haya yanahitajika kuwa ya kina na kibinafsi. Kwakuwa utafiti huu ni wa siri, hakuna mtu atajua majibu yako. Tungependa uhusianio wako wa kina kwa kujibu maswali haya. Kama kuna swali unalosikia hautaki kulijibu, tafadhali nijulishe ili tuliache.*			
6.1	**Kwa sasa uko na mpenzi?**	01Ndio 02 La 88 DK 99 NR	
6.2	**Umewahi kufanya ngonoya kupitia kuma au kupitia mkunduni?**<br>NOTE: Hii ni ngono ya kutaka ama ngono ya kulazimishwa.	01 Ndio 88 DK→<br>02 La → 99 NR→	
6.3	**Ulikuwa na umri gani ulipofanya ngono kupitia kuma au mkunduni kwa mara ya kwanza?**	Miaka |___||___| 88 DK 99 NR	
6.4	**Katika miezi 6 iliyopita umewahi fanya ngono kupitia kuma au kupitia mkunduni?**	01 Ndio 02 La 88 DK 99 NR	
Idadi ya wapenzi hutofautiana sana kutoka mtu moja hadi mwingine. Watu wengine husema kuwa wana mpenzi mmoja, wengine wawili, na wengine kufikia wapenzi kwa mamia.			
6.5A	**Katika kipindi cha miezi 6 iliyopita, niwatu wangapi uliofanya ngono nawao?**	|___||___| |___||___| 88 DK 99 NR	
6.5B	**Katika mwaka mmoja uliopita niwatu wangapi uliofanya ngono nawao?**	|___||___| |___||___| 88 DK 99 NR	
6.6	**Katika kipindi cha miezi 6 iliyopita ni mara ngapi uli tumia mipira na wa/mshirika wako wa ngono wa mara kwa mara?**<br>NOTE: Mshirika wa mara kwa mara ni mtu yeyote unashirikiana kimapenzi kila wakati .	01 Sina mshirika wa mara kwa mara<br>02 Kila wakati<br>03 mara nyingi<br>04 wakati mwingine<br>05 mara chache<br>06 Hatujatumia Kamwe 88 DK 99 NR	
6.7	**Katika mwezi uliopita ni mara ngapi uli tumia mipira na wa/mshirika wako wa ngono wa mara kwa mara??**<br>NOTE: Mshirika wa kawaida ni mtu/watu unaoshirikiana kimapenzi mara moja na sio mteja wa kulipa.	01 Sina mshirika wa kawaidia<br>02 Kila wakati<br>03 mara nyingi<br>04 wakati mwingine<br>05 mara chache<br>06 Hatujatumia Kamwe 88 DK 99 NR	

ID: __________-___
Tarehe: ___/____/_____

6.8	**Katika mwezi uliopita , ni mara ngapi uli tumia mipira ya kondomu wakati wa ngono ya kulipwa.**	01 Sija fanya ngono yakulipwa<br>02 Kila wakati<br>03 Mara nyingi<br>04 Wakati mwingine<br>05 Mara chache<br>06Sijatumia Kamwe 88 DK 99 NR				
6.9	**Ulitumia mipira ya kondomu kila wakati wa ngono katika miezi 6 iliyopita ?**	01 Ndio 02 La 88 DK 99 NR				
6.10	**katika kipindi cha miezi 6 iliyopita , umewahi kupata ugonjwa wowote kupitia ngono?**	01 Ndio 02 La 88 DK 99 NR				
6.11	**Umewahi fanya ngono ukiwaumelewa kwa pombe?**	01 Ndio 02 La 88 DK 99 NR				
6.12	**Umewahi fanya ngono ukiwa umelewa kwa dawa za kulevya?**	01 Ndio 02 La 88 DK 99 NR				
6.13	**Je, unatumia (au mpenzi wako) njia za upangaji wa uzazi ili kuzuia mimba?**	01 Ndio 02 La 88 DK 99 NR				
6.14A	**Ni njia gani ya upangaji wa uzazi unayoitumia kwa sasa?<br>(chagua zote zinazo tumika)**	01 Tembe za kila siku<br>02 Njia ya Sindano<br>03 Mipira ya Kondomu<br>04 Dawa za kuua mbegu za kiume<br>05 Kifaa cha kuwekwa kwa kizazi cha mwanamke<br>06 Kifaa cha kudungwa mwilini / Diaphram<br>07 Kuchukua muda bila ngono<br>08 Withdrawal<br>09 Njia zingine za kitamaduni<br>10 Kutolewa mbegu au kizazi cha kiume au kike88 DK 99 NR				
6.14B	**Ni sababu gani hautumii njia ya kupanga uzazi kwa sasa?<br>(chagua zote zinazo tumika)**	01 Sina hisia za ngono<br>02 Gharama ya kununua<br>03 Sina uhakika ambapo nitaipata<br>04 Aibu<br>05 Wasiwasi juu ya madhara<br>06 Mpenzi au familia kuzuia au kukataa<br>07 Nyinginezo (Taja):________________________<br>88 DK 99 NR				
6.15	**Umewahi kufanya ngono na mtu yeyote ambao humjui (stranger).**	01 Ndio 02 La 88 DK 99 NR				
	**Swali**	**Jibu**				**Ruka**
6.16	**Kwa kipindi cha miezi 6 iliyopita, umewahi fanya ngono kwajili kupata au kupewa hizi?**	01<br>Ndio	02<br>La	88<br>DK	99<br>NR	
6.16A	Badala ya chakula					
6.16B	Badala ya nyumba/makao					
6.16C	Badala ya fedha					
6.16D	Badala ya madawa au dawa za kulevia					
6.16E	Badala ya vitu vinginevyo (Taja):<br>____________________________					
6.17	**Ni mara ngapi umewahi fanya ngono na mtu yeyote kupata moja ya mambo haya?<br>(Chagua moja)**	01 Kamwe sijafanya<br>02 Mara Moja<br>03 Mara 2 au 3<br>04 Mara 4 nakuzidi 88 DK 99 NR				
6.18	**Kwa kipindi cha miaka 2, umewahi pewa fedha zaidi kwa ajili kushirikiani kingono bila kutumia mipira ya kondomu?**	01 Ndio 02 La 88 DK 99 NR				
6.19	**Kwa kipindi cha miaka 2, umewahi kubali fedha zaidi kwa ajili kushirikiana kingono bila kutumia mpira ya kondomu?**	01 Ndio 02 La 88 DK 99 NR				
Sasa, Ninataka ufikirie mpenzi wako wa mwisho wa hivi karibuni.						

6.20	**Mpenzi wako ana umri gani?**	Miaka |___||___| 88 DK 99 NR	
6.21	**Mpenzi wako wa hivi karibuni ni wa jinsia ipi?**	01 Mwanamume<br>02 Mwanamke<br>03Nyinginezo (Taja): ____________________	
6.22	**Una uhusiano gani na mpenzi huyu wako? (Chagua moja)**	01 Mpenzi mkuu<br>02 Mpenzi wa mara kwa mara<br>03 Mpenzi wa kawaida/ mara moja<br>04 Mteja wa ulipia ngono<br>05 Nyinginezo (Taja:)____________________	
6.23	**Mara ya mwisho uliposhirikiana kingono na mpenzi wako wa hivi karibuni, mlitumia mpira wa kondomu?**	01 Ndio 02 La 88 DK 99 NR	
6.24	**Umewahi ongea na huyu mtu kuhusu kutumia mipira ya kondomu?**	01 Ndio 02 La 88 DK 99 NR	
6.25	**Umewahi ongea na huyu mtu kuhusu hatari za kibinafsi za kuambukizwa na virusi vinavyosababisha ukimwi?**	01 Ndio 02 La 88 DK 99 NR	
6.26	**Umewahi ongea na huyu mtu kuhusu kujikinga na kuambukizwa na virusi vinavyosababisha ukimwi?**	01 Ndio 02 La 88 DK 99 NR	
6.27	**Umewahi kumuliza mtu huyu kutumia mipira ya kondomu?**	01 Ndio 02 La 88 DK 99 NR	
6.28	**Unapokea msaada wa kifedha mara kwa mara kutoka kwa huyu mtu?**	01 Ndio 02 La 88 DK 99 NR	
6.29	**Katika kipindi cha miezi 6 iliyopita, umewahi lipa huyu mtu kwa kifedha au bidhaa nyingine badala ya ngono?**	01 Ndio 02 La 88 DK 99 NR	
6.30	**Katika kipindi cha miezi 6 iliyopita, umewahi lipwa na huyu mtu kwa kifedha au bidhaa nyingine badala ya ngono?**	01 Ndio 02 La 88 DK 99 NR	
6.31	**Katika kipindi cha miezi 6 iliyopita, huyu mtu ameshirikiana kwa ngono na mtu mwingine licha wewe?**	01 Ndio 02 La 88 DK 99 NR	
6.32	**Mtu huyu amewewahi kukupiga kofi?**	01 Ndio 02 La 88 DK 99 NR	
6.33	**Mtu huyu amewahi kukupiga kwa ngumi au kifaa kingine kinachoweza kuumiza?**	01 Ndio 02 La 88 DK 99 NR	
6.34	**Umewahi jisikia umeshirikiana kingono na mtu huyu kwa ajili ya kupata fedha, chakula au makao?**	01 Ndio 02 La 88 DK 99 NR	
6.35	**Umewahi jisikia umeshirikiana kingono na mtu huyu kwa ajili ya kupata mapato ya aina ya kifedha?**	01 Ndio 02 La 88 DK 99 NR	
Sasa, Ninataka ufikirie mpenzi wako aliyekuwa kabla ya huyu wa mwisho wa hivi karibuni.Tutamuita mpenzi wa pili.			
6.36	**Umekuwa na wapenzi wengine kabla huyu wa hivi karibuni?**	01 Ndio 88 DK<br>02 La 99 NR	
6.37	**Mpenzi wako wa pili ana miaka mingapi?**	Miaka |___||___| 88 DK 99 NR	
6.38	**Mpenzi wako wa pili ni wa jinsia ipi?**	01 Mwanamume<br>02 Mwanamke<br>03Nyinginezo (Taja): ____________________	
6.38	**Ulikuwa na uhusiano gani na mpenzi huyu wapilii? (Chagua moja)**	01 Mpenzi mkuu<br>02 Mpenzi wa mara kwa mara<br>03 Mpenzi wa kawaida/ mara moja<br>04 Mteja wa ulipia ngono<br>05 Nyinginezo (Taja:) ____________________	
6.39	**Mara ya mwisho uliposhirikiana kingono na mpenzi wako wa pili, mlitumia mpira wa kondomu?**	01 Ndio 02 La 88 DK 99 NR	
6.40	**Umewahi ongea na huyu mtu kuhusu kutumia mipira ya kondomu?**	01 Ndio 02 La 88 DK 99 NR	
6.41	**Umewahi ongea na huyu mtu kuhusu hatari za kibinafsi za kuambukizwa na virusi vinavyosababisha ukimwi?**	01 Ndio 02 La 88 DK 99 NR	
6.42	**Umewahi ongea na huyu mtu kuhusu kujikinga na kuambukizwa na virusi vinavyosababisha ukimwi?**	01 Ndio 02 La 88 DK 99 NR	
6.43	**Umewahi kumuliza mtu huyu kutumia mpira wa kondomu?**	01 Ndio 02 La 88 DK 99 NR	

6.44	**Unapokea msaada wa kifedha mara kwa mara kutoka kwa huyu mtu?**	01 Ndio 02 La 88 DK 99 NR	
6.45	**Katika kipindi cha miezi 6 iliyopita, umewahi lipa huyu mtu kwa kifedha au bidhaa nyingine badala ya ngono?**	01 Ndio 02 La 88 DK 99 NR	
6.46	**Katika kipindi cha miezi 6 iliyopita, umewahi lipwa na huyu mtu kwa kifedha au bidhaa nyingine badala ya ngono?**	01 Ndio 02 La 88 DK 99 NR	
6.47	**Katika kipindi cha miezi 6 iliyopita, huyu mtu ameshirikiana kwa ngono na mtu mwingine licha wewe?**	01 Ndio 02 La 88 DK 99 NR	
6.48	**Mtu huyu amewewahi kukupiga kofi?**	01 Ndio 02 La 88 DK 99 NR	
6.49	**Mtu huyu amewahi kukupiga kwa ngumi au kifaa nyingine inayoweza kuumiza?**	01 Ndio 02 La 88 DK 99 NR	
6.50	**Umewahi jisikia ya kuwa umeshirikiana kingono na mtu huyu kwajili ya kupata fedha, chakula au makao?**	01 Ndio 02 La 88 DK 99 NR	
6.51	**Umewahi jisikia ya kuwa umeshirikiana kingono na mtu huyu kwajili ya kupata mapato ya aina ya kifedha?**	01 Ndio 02 La 88 DK 99 NR	

Section 7: Juhudi za kukinga virusi vinavyosababisha ukimwi[O]
Maswali yafuatayo ni ya binafsi na yanauliza kuhusu husiano wako na historia ya kujamiiana. Kama kuna swali unayosikia hautaki kuijibu, tafadhali nijulishe ili tuliache

No.	Swali	Jibu	Ruka
7.1	**Kwa maoni yako, vijana katika makazi haya huzuia kuenea kwa virusi vinavyosababisha ukimwi kwa njia gani?**<br>**(Chagua zote zinazofaa)**	01 Kujiepusha na ngono<br>02 Kutimia mipira za kondomu<br>03 Kutima mipira ya kondomu kwa vijana wanojihusisha na ngono kwa mara ya kwanza<br>04 Badili mienendo kwa kuchagua wapenzi<br>05 Punguza idadi ya wapezi<br>06 Ongea na wa/mpenzi kuhusu virusi HIV<br>07 Ongea juu ya mipira ya kondomu na wa/mpenzi<br>08 kuwa na mpenzi mmoja pekee<br>09 Tumia dawa za PrEP (Pre-exposure prophylaxis)<br>10 Tumia dawa za PEP (Post-Pre-exposure prophylaxis)<br>11 Tumia chanjo za HIV na microbicides<br>12 Tohara ya Hiari kwa manamume<br>13Nyinginezo (Taja)*(_______________________)*<br>88 DK 99 NR	
7.2	**Ni nini umefanya ili kuzuia na/au kupunguza hatari ya kuambukizwa na virusi vinavyo sababisha ukimwi?**<br>(NOTE: Usisome majibu ya hii karatasi, tia alama kwa majibu zote anayehojiwa anataja	01 Kujiepusha na ngono<br>02 Kutimia mipira za kondomu<br>03 Kutima mipira ya kondomu kwa vijana wanojihusisha na ngono kwa mara ya kwanza<br>04 Badili mienendo kwa kuchagua wapenzi<br>05 Punguza idadi ya wapezi<br>06 Ongea na wa/mpenzi kuhusu virusi HIV<br>07 Ongea juu ya mipira ya kondomu na wa/mpenzi<br>08 kuwa na mpenzi mmoja pekee<br>09 Tumia dawa za PrEP (Pre-exposure prophylaxis)<br>10 Tumia dawa za PEP (Post-Pre-exposure prophylaxis)<br>11 Tumia chanjo za HIV na microbicides<br>12 Tohara ya Hiari kwa manamume<br>13 Nyinginezo (Taja) *(_______________________)*<br>88 DK 99 NR	

ID: __________-___
Tarehe: ___/____/_____

7.3	**Katika kipindi cha miezi 6 iliyopita, umewahi nunu mipira ya kondomu na fedha zako zenyewe?**	01 Ndio 02 La 88 DK 99 NR				
7.4	**Katika kipindi cha miezi 6 iliyopita, ni fedha ya kiasi gani umetumia kunua mipira ya kondomu?**	[___|___][___|___][___|___] Ksh 88 DK 99 NR				
7.5	**Kwa kipindi cha mwaka mmoja iliyopita umewahi pata mipira ya kondomu ya bure?**	01 Ndio 02 La 88 DK 99 NR				
7.6	**Ulitumia mipira ya kondomu uliyo pewa?**	01 Ndio 02 La 88 DK 99 NR				
	**Swali**	**Jibu**				
7.7	**Tafadhali ni julishe ni mbinu gani ulizozifuta ndani ya kipindi cha miezi 6 iliyopita, kwasababu ulidhani zitakuepusha kuambukizwa au kuambukiza virusi vinanvyosababisha ukimwi?**	Ndio, Nilifanya na ikafanya kazi. 01	Ndio Nilijaribu, lakini haikufanya kazi. 02	La, Sija jaribu lakini inawezekan a 03	La, Sijajaribu lakini haiwezekani 04	88 DK 99 NR
7.7A	Tumia mipira ya kondomu kilawakati wa ngono					
7.7B	Tumia mipira ya kondomu wakati mwingine wa kufanya ngono					
7.7C	Kufanya ngono na mtu mmoja pekee					
7.7D	Kufanya ngono na mtu mmoja pekee na kumuliza juu ya hali yake ya kiafia kuhusiana na virusi vyakusababisha ukimwi					
7.7E	Kufanya ngono na mtu mmoja asiye na virusi vyakusababisha ukimwi na hana wapenzi wengine.					
7.7F	Kupimwa kwa virusi vyakusababisha ukimwi kujua hali yangu yakiafia					
7.7G	Kuuliza mpenzi wangu kupimwa kwa virusi vyakusababisha ukimwi					
7.7H	Kuongea namna tunaweza epuka kuambukizwa virusi vya kusababisha ukimwi na mpenzi wangu					
7.7I	Meza dawa(PreP) za kuzuia kuambukizwa virusi vyakusababisha ukimwi kabla ya ngono					
7.7J	Meza dawa(PeP) za kuzuia kuambukizwa virusi vyakusababisha ukimwi baadaya ya ngono (PeP)					
7.7K	Meza dawa (ART)kila siku zakupunguza idadi ya virusi vya kusababisha ukiwmi mwilini					
7.7L	Tumia madawa ya topical microbicides au za kuzuia kuambukiza kabla au baadaya ngono (PeP/Prep)					
7.7M	Badilisha aina ya mpenzi niko naye					
7.7N	Punguza idadi ya watu ninao jihusiha nao kwa njia ya ngono					
7.7O	Kaa bila ngono kwa miezi 6					
7.7P	Tumia microbicides					
7.7Q	Kupitia tohara ya kiuume hospitalini					
	**Swali**	**Jibu**				**Ruka**
7.8	Kwa maoni yako, motisha ya kifedha inawezekano wa kuwahamasisha kupitisha juhudi za kuzuia kuambkukizwa kwa virusi vinavyosababisha ukimwi kwa vijana kwa makazi yako?	01 Ndio 02 La 88 DK 99 NR				
7.9	Kiasi gani ya fedha kila mwezi inaweza kuwa motisha kwa mradi huu kuwa na ufanisi?	|___||___| |___||___|ksh 88 DK 99 NR				

7.10	Kiasi gani ya fedha kila mwezi inaweza kuwa motisha kwa mradi huu kuwa na ufanisi wa kiasi?	|___||___| |___||___|ksh 88 DK 99 NR	
7.11	Kiasi gani ya fedha kila mwezi inaweza kuwa motisha kwa mradi huu kuwa na ufanisi kidogo?	|___||___| |___||___|ksh 88 DK 99 NR	

Section 8: Kutafuta huduma za matibabu zinazohusu virusi vinavyosababisha Ukimwi [O]
Maswali yafuatayo ni ya binafsi na yanauliza kuhusu husiano wako na historia ya kujamiiana. Kama kuna swali unayosikia hautaki kuijibu, tafadhali nijulishe ili tuliache.

No.	Swali	Jibu	Ruka
8.1	**Umewahi kupimwa kwa virusi vya Ukimwi?**	01 Ndio 02 La 88 DK 99 NR	
8.2	**Ni miezi ngapi iliyopita tangu upimwe kwa virusi vya ukimwi mwisho?**	Miezi [___|___] 88 DK 99 NR	
8.3	**Ilikuwa mara yako ya kwanza kupimwa kwa virusi vya ukimwi?**	01 Ndio 02 La 88 DK 99 NR	
8.4	**Umepimwa kwa virusi vya ukimwi mara ngapi maishani mwako?**	[___|___] Idadi ya mara88 DK 99 NR	
8.5	**Wakatiwamwisho kupimwa kwa virusi vya ukimwi, ulimwonyesha mtu mwingine matokeo yako?**	01 Ndio 02 La 88 DK 99 NR	
8.6	**Iwapo ulipatikana au haukupatikana na virusi vya ukimwi, ni nani umemwambia matokeo yako?**<br>**(Chagua zote zinazofaa)**	01 Mpenzi wa ngono<br>02 Rafiki<br>03Familia<br>04 Muajiri<br>05Mtaalamu/Mhudumu wa afya<br>06Nyinginezo (taja): ____________________	
8.7	**Ni wapi mwisho ulipimwa kwa virusi vya ukimwi?**<br>NUKULI :Mifano ya mazingira ya kliniki ni ofisi binafsi ya daktari, hospitali, kliniki ya afya ya umma au kituo cha afya ya jamii. Mifano ya mazingira zisizo kliniki ni pahala pa ushauri nasaha na kupima kwa virusi vya ukimwi, mipango kuwafikia mitaaniau nyumbani.	01 Mazingara ya Kliniki/Hospitali<br>02Maingara zisizo ya Kliniki/Hospitali<br><br>88 DK 99 NR	
8.8	**Ulitumia kiasi gani cha fedha kwa kila moja ya yafuatayo?**		
8.8A	Ada kwa ajili ya huduma ya kupimwa kwa virusi vya ukimwi.	[___|___][___|___][___|___] Ksh 88 DK 99 NR	
8.8B	Ada nyingine katika kliniki kuhusiana na kupimwa kwa virusi vya ukimwi.	[___|___][___|___][___|___] Ksh 88 DK 99 NR	
8.8C	Nauli ya usafirishaji mpaka pahala pa kupimwa virusi vya ukimwi	[___|___][___|___][___|___] Ksh 88 DK 99 NR	
8.8D	Chakula ulichonunua ukisafiri mpaka pahala pa kupimwa virusi vya ukimwi	[___|___][___|___][___|___] Ksh 88 DK 99 NR	
8.8E	Kulipia huduma ya watoto	[___|___][___|___][___|___] Ksh 88 DK 99 NR	
8.8F	Gharama nyingine (taja):______________________	[___|___][___|___][___|___] Ksh 88 DK 99 NR	
8.9	**Ulitumia masaa mangapi kutoka mwanzo hadi mwisho kupata huduma ya kupimwa kwa virusi vya ukimwi? (taja pamoja na muda wa kusafiri na kusubiri)**	Masaa [___|___]88 DK 99 NR	
8.10	**Ni kiasi gani ya fedha ungepata katika hizo masaa ulizotumia kupimwa kwa virusi vya ukimwi?**	[___|___][___|___][___|___] Ksh 88 DK 99 NR	

8.11	**Katika miezi 3 ijayo, unakusudia kutumia yoyote ya huduma zifuatazo kwa masuala yanayohusiana na virusi vya ukimwi au ukimwi? (Chagua zote zinazofaa)**	01 Vituo vya kuelmisha juu wa Ukimwi<br>02 Ushauri wa virusi vya ukimwi na Ukimwi<br>03 Kupima virusi vya ukimwi<br>04 Kikundi ya kusaidina waathirika wa virusi vya ukimwi na ukimwi<br>05 Waganaga na madawa ya kienyeji<br>06 huduma za afia zisizo za kupimia virusi vya ukimwi<br>07 Usaidizi kutoka kwa raia<br>08 Huduma za kisehria<br>09 Zinginezo (taja): _______________88 DK 99 NR	
8.12	**Katika miezi 12 iliyopita, umekuwa na mazungumzo ana kwa ana na mfanayakazi wa uhamasishajiau mshauri kuhusu njia za kuzuia virusi vya ukimwi?**<br>**NOTE: Hii haihusishi mazungumzo wakati wa kupimwa kwa virusi vya ukimwi.**	01 Ndio 02 La 88 DK 99 NR	
8.13	**Katika kipinda ya miezi 12 iliyopita, umeshirikiana katika majadiliano rasmi kwa vikundi iliyo pangwa kwa kikao juu ya njia ya kuzuia virusi vya ukimwi?**<br>NOTE: Hii haihusishi majadiliano yasio rasmi katika marafaki.	01 Ndio 02 La 88 DK 99 NR	
8.14	**Umewahi sikia juu ya kujipima kibinafsi virusi vya ukimwi kutumia njia ya mdomoni?**<br>NOTE: Njia hii ya kujipima virusi vya ukimwi, inaweza kufanyika nyumbani kwa kupima mate na kupata majimbu baada ya dakika ishirini.	01 Ndio 02 La 88 DK 99 NR	
8.15	**Unaweza kuwa tayari kutumia njia hii ya kujipima virusi vya ukimwi siku zijazo?**	01 Ndio 02 La 88 DK 99 NR	
8.16	**Kama NDIO,sababu yakuchagua njii ya kujipima virusi vya ukimwi ni ipi? (Chagua zote zinazofaa)**	01 Kujua hali ya afia kuhusu virusi vya ukimwi<br>02 Kuongeza kwa Siri<br>03 Kuongeza kwa urahisi<br>04 Uhuru wa kujipima mwenyewe<br>05 Kupunguza unyanyapaa<br>06 Kutojulikana hali ya kiafia<br>07 Kupunguza muda unotumia<br>08 Kupunguza utumiaji wa pesa<br>09 Urahisi wa kurudia kujipiima<br>10 Kupatikana virahisi kwa vitua vingi<br>11 Zinginezo (Taja): ______________________<br>88 DK 99 NR	
8.17	**KamaLA, sababu yaku kata kutumia njii ya kujipima virusi vya ukimwi ni ipi? (Chagua zote zinazofaa)**	01 Gharama ya juu<br>02 Wezekano wa majibu isiyo sawa<br>03 Ukosefu wa ushauri<br>04 Ugumu wa rufaa na kupata matibabu<br>05 Kituo cha kupimia virusi vya ukimwi ni bure<br>06 Usalama wa kutupa kifaa ya kujipimia<br>07 Uwezekano wa kujidhuru kibinafsi<br>08 Kutumika kwa kutumia nguvu<br>09 matumizi isiyofaa ya fedha<br>10 Kuendeleza uzembeaji wa kufanya ngono bila kujikinga<br>11 Zinginezo (Taja): _____________________<br>88 DK 99 NR	

8.18	**Una weza kuwa sawa kuulizia wa/mpenzi wako kutumia njia ya kujipima virusi vya ukimwi kabla ya ngono?**	01 Ndio 02 La 88 DK 99 NR	
8.19	**Ungependelea kuenda wapi kuchukua vifaa vya kujipimia virusi vya ukimwi?**<br>**(Chagua moja)**	01 Kitupo chakupimia virusi vya ukimwi<br>02 Hospitali<br>03 Maabara au duka la dawa<br>04 Kituo cha Jamii<br>05 Shuleni<br>06 Pahali pa Kazi<br>07 Nyinginezo (Taja): __________ 88 DK 99 NR	
8.20	**Kwa sasa unaweza pata simu ya rununu?**	01 Ndio 02 La 88 DK 99 NR	
8.21	**Unasaidia mtu mwengine akitaka kutumia simu yako ya rununu?**	01 Ndio 02 La 88 DK 99 NR	
8.22	**Baada ya kujipima virusi vya ukimwa mwenyewe, utataka kupata ushauri kupitia simu yako ya rununu?**	01 Ndio 02 La 88 DK 99 NR	
8.23	**Unaweza kupata mtandaokupitia simu yako ya rununu?**	01 Ndio 02 La 88 DK 99 NR	
8.24	**Umewahi pata ujumbe mfupi kupitia simu yako ya rununu?**	01 Ndio 02 La 88 DK 99 NR	
8.25	**Umewahi pata ujumbe mfupi kupita simu yako ya rununu, kuhusiana na kuzuia virusi vya ukimwi?**	01 Ndio 02 La 88 DK 99 NR	
8.26	**Umewahi pata ujumbe mfupi kupita simu yako ya rununu, kuhusiana na tiba na matunzo ya virusi vya ukimwi?**	01 Ndio 02 La 88 DK 99 NR	
8.27	**Umewahi pata ujumbe mfupi kupita simu yako ya rununu, kuhusiana nakujiendeleza kifedha**	01 Ndio 02 La 88 DK 99 NR	
8.28	**Katika mwezi mmoja, unatumia fedha kiasi gani kuweka moto kwenye batari na kutumia kwa kuongea?**	[___|___][___|___][___|___] Ksh 88 DK 99 NR	

**Section 9: Tabia za Kiuchumi**		
9.1	**Wewe ni mshindi wa tuzo na unatakikana kuchagua kuchukua shilingi 50,000($500) kesho au shilingi 75,000($750) kwa mwezi moja .Utachagua ipi?** (Chagua moja)	01 50,000 KSH kesho<br>02 75,000 KSH kwamwezi moja<br>88 DK 99 NR
9.2	**Baadaye unatuzwa shilling 100,000 ($1,000 USD) badaya ya mwezi moja kutoka leo? Ungependelea ipi?**(Chagua moja)	01 50,000 KSH kesho<br>02 100,000 KSH kwamwezi moja<br>88 DK 99 NR
9.3	**Badala ya shilingi 60,000 Ksh($600 USD) ulituzwa na kuchukua kesho, ungependelea ipi?** (Chagua moja)	01 60,000 KSH kesho<br>02 75,000 KSH kwamwezi moja<br>88 DK 99 NR
9.4	**Tuseme ulikuwa mshindi wa tuzo, ungechagua kupewa shilingi 500 ($5 USD)kesho au shilingi 1000 Ksh ($10 USD) kwa mwaka moja?** (Chagua moja)	01 550 KSH kesho<br>02 1,000 KSH kwa mwaka moja<br>88 DK 99 NR
9.5	**Ukiwa na shilling 10,000 ($100USD)leo,ungeitumia kivipi?** (chagua moja)	01 Tumia fedha zote<br>02 Tumia fedha karibu ya zote<br>03 Tumia nusu, weka akiba nusu<br>04 Weka akiba karibu fedha zote<br>05 Weka akiba fedha zote 88 DK 99 NR
9.6	**Unajua mtu yeyote wa karibu anayeishi na virusi vya ukimwi?**	01 Ndio 02 La 88 DK 99 NR
9.7	**Unajua mtu yeyote ambaye amefaidika kwa kutumia madaya ya kupunguza kiwango cha virusi vya ukimwimwilini(ARV)?**	01 Ndio 02 La 88 DK 99 NR
9.8	**Ulisikia juu ya haya faidia hii katika kipindi ya mwaka moja iliyopita au zaidi?**	01 Katika kipindi cha mwaka moja<br>02 Kupitia mwaka moja 88 DK 99 NR

9.9	**Unajua mtu yeyote amenufaika kwa kujua hali yake ya kiafia kulingana na virusi vya ukimwi, kupitia kwa vituo vya kupimia na ushauri wa hiari?**	01 Ndio 02 La 88 DK 99 NR
9.10	**Ulisikia juu ya faida hii katika kipindi ya mwaka uliyopita au kupita?**	01 kipindi cha mwaka jana<br>02 Kupita mwaka jana 88 DK 99 NR
9.11	**Unajua rafiki wa karibu au familia aliyefariki kutokana na maradhi ya ukimwi?**	01 Ndio 02 La 88 DK 99 NR
9.12	**Mtu huyo alifariki katika kipindi ya mwaka uliyopita au kupita?**	01 kipindi cha mwaka jana<br>02 Kupita mwaka jana88 DK 99 NR
9.13	**Unajua mtu yeyote kwenye nyumba yako aliye pimwa virusi vya ukimwi?**	01 Ndio 02 La 88 DK 99 NR
9.14	**Kwa ufahamu wako, kupimwa kwao virusi vya ukimwi, ulikuwa katika kipindi ya mwaka uliyopia au mwaka mmoja uliyopita?**	01 kipindi cha mwaka jana<br>02 Kupita mwaka jana88 DK 99 NR
	**Swali**	**Jibu**
9.15	**Jinsi gani unavyo weza kujipima: Tafadhali tumia kiwango kutoka 0 hadi 10, wapi 0 inamanisha "hainielezei kikamilifu"na 10 inamanisha "Inanielezea kikamilifu"**	
9.15A	Huwa nina chukua tahadhari	Hainielezei kikamilifu Inanielezea kikamilifu<br>0 1 2 3 4 5 6 7 8 9 10
9.15B	Huwa nina hairisha vitu hata kama ni bora kuvifanya na kumalizia papo hapo.	Hainielezei kikamilifu Inanielezea kikamilifu<br>0 1 2 3 4 5 6 7 8 9 10
9.15C	Najinyima vitu vingine leo ili iniwezeshe kujimudu kimaisha kesho.	Hainielezei kikamilifu Inanielezea kikamilifu<br>0 1 2 3 4 5 6 7 8 9 10
9.16	**Ukiweza kushinda tuzo katika mchezo. Ungependelea kupata ipi?**(Chagua moja)	01 Shilingi 5,000 kwa hakika<br>02 Nafasi ya kuchezea 1 hadi 10 ili kushinda shilingi 40,000<br>03 Nafasi ya kuchezea 1 hadi 100 ili kushinda shilingi300,000<br>88 DK 99 NR
9.17	**Ulicheza mchezo wa bahati na ulipoteza. Ungependelea kupoteza ipi?**(Chagua moja)	01 Shilingi 5,000 kwa hakika<br>02 Nafasi ya kuchezea 1 hadi 10 nakupoteza shilingi 40,000<br>03 Nafasi ya kuchezea 1 hadi 100 nakupoteza shilingi 300,00088 DK 99 NR

**Section 10. Familia na ushirikiano wa kichumi**			
**No.**	**Swali**	**Jibu**	**Ruka**
10.1A	**Familia yako iko na akiba yoyote ya kifedha?**	01 Ndio 02 La 88 DK 99 NR	
10.1B	**Kama ndio, ni kiwango kipi cha akiba ya kifedha?**	|___||___| |___||___| ksh 88 DK 99 NR	
10.2A	**Katika kipindi cha mwaka moja uliopita, famila yako ilikopa fedha kutoka taasisi yoyote?**	01 Ndio 02 La 88 DK 99 NR	
10.2B	**Kama ndio, mlikopa fedha kiasi kipi?**	|___||___| |___||___| ksh 88 DK 99 NR	

Section 10. Familia na ushirikiano wa kichumi			
**No.**	**Swali**	**Jibu**	**Ruka**
10.2C	**Familia yako ilitumia mkopo huu kwa kufanyia nini?**	01 Kuanzisha Biashara<br>02 Gharama ya Nyumbani<br>03 Gharama ya Matibabu<br>04 Gharama ya Elimu<br>05 Nyinginezo(Taja):____________<br>88 DK 99 NR	
10.3A	**Kuna mmoja wa familia yako aliyekopa fedha kwa mtu binafsi na kuhitajika kulipa?**	01 Ndio 02 La 88 DK 99 NR	
10.3B	**Kama ndio, anadaiwa fedha kiwango kipi ilikusawazisha deni hili? ?**	|___||___| |___||___|ksh 88 DK 99 NR	
10.3C	**Umewahi kopa fedha kwa watu wenigine ilikugharamia matibabu yakiafia yako au ya familia yako?**	01 Ndio 02 La 88 DK 99 NR	
10.4	**Mwezi ukiyopita, familia yako iliwahi kupata usaidizi wowote wa kimali, (i.e., chakula, nguo, vifaa vingine) kutoka mtu yeyote asie wa familia yako?**	01 Ndio 02 La 88 DK 99 NR	
10.5	**Familia yako iliwahi kopesha watu wengine fedha? Kama ndio, kiasi gani?**	01 Ndio 02 La 88 DK 99 NR	
10.6	**Mwezi uliopita, familia yako iliwahi kupata mapato ya fedha ya kiasi gani kwa ki ujumla?**	KSH 25,000 na kupungua……………01<br>KSH 25,001 – 50,000……………..02<br>KSH 50,001 – 75,000……………..03<br>KSH 75,001 – 100,000……………04<br>KSH 100,001 – 150,000……..…...05<br>KSH 150.001 – 200,000…………..06<br>KSH 200,001 – 300,000………..….07<br>KSH 300,001 – 400,000…………..08<br>KSH 400,001 – 500,000…………..09<br>KSH 500,001 au kuzidi…………10<br>88 DK 99 NR	
10.7	**Ni nani anayemiliki nyumba unayoishi ?**<br>**(Chagua moja)**	01 Mimi mwenyewe<br>02 Mzazi au Mlezi<br>03 Familia au Rafiki<br>04 Ya kukodeshawa<br>05 Isiyo kodeshwa na kumilikiwa na mtu<br>06Nyinginezo (taja): _______________	
10.8	**Ukifikiria mapato ya ki ujumla ya familia yako, unaweza sema kwamba munaweza kujikimu virahisi kimasha?**<br>**(Chagua moja)**	01 Virahisi sana<br>02Virahisi kiasi<br>03Sio virahisi wala vigumu<br>04 Kwa vigumu kiasi<br>05Kwa vigumu sana<br>88 DK 99 NR	
10.9	**Kumalizia sehemu hii, kwa mmoja ujao kutoka leo, unaona familia yako itakuwa inaweza au kutoweza kuishi na kujikimu virahisi kuliko hivi sasa?**	01 Tutaishi vizuri kuliko sasa<br>02 Haitakuwa na mabadilkio<br>03 Tutaishi vibaya kuliko sasa<br>88 DK 99 NR	
10.10	**Fikiria ngazi iliyio na hatua 9,Hatua ya chini ya kwanza ndipo watu wasiojiweza wapo, na hatua ya juu kabisa ndipo watuwanaojiweza wapo. Familia yako inasimamia hatua gani kwenye ngazi hii kwa leo?**	Wasiojiweza Waojiweza<br>1 2 3 4 5 6 7 8 9	

		01 - Ndio	02- La	88-DK	99-NR	Ruka

Section 10. Familia na ushirikiano wa kichumi						
**No.**	**Swali**	**Jibu**				**Ruka**
10.11	**Makao yako ikona vitu vyovyote vifuatazo following?**					
10.11A	Maji ya mfereji					
10.11B	Umeme/Electricity					
10.11C	Runinga/Tv					
10.11D	Jiko la Umeme au Gesi					
10.11E	Redio					
10.11F	Simu ya rununu					
10.12A	**Asante kwakutupa muda wako na kujibu maswali ya utafiti huu.<br>Swali langu la mwisho nikuhusu usalama wako kutokanana na kushirikiana kwa utafiti huu.<br><br>Ulipata kutishwa kutokanana na kushirikiana kwa utafiti huu?**	01 – Ndio 02 – La 88 DK 99 NR				
10.12B	**Una tarajia kutishwa baadaye kutokanana na kushiriki kwa utafiti huu?**	01 – Ndio 02 – La 88 DK 99 NR				

HUU NDIYO MWISHO WA MAHOJIANO HAYA
Asante kwa kuchukuwa muda wako kuongea namimi leo.
Je, uko na maono yakuongezea au maswali yoyote ungependelea kuuliza?

KUJAZWA NA ALIYEHOJI :

11.1	Tafadhali nuku hali ya kukamilika kwa mahojiano haya.	01 – Kukamilika<br>02 – Haijakamilika
11.2	Sababu ya kutomaliza mahojiano?	01 – Masaa hayakutosha<br>02 –Kusumbuliwa kila wakati<br>03 – Mshiriki alifadhaika<br>04 - Mshiriki alikuwamame choka<br>05 – Mshiriki ali ulizia kumaliza kabla<br>88 DK 99 NR
11.3	Mshiriki alikubali kuwashirikisha wenzake wengine warika yake?	
11.4	Ni sababu gani kukata kuwashirikisha watu warika yake wengine?	01 – Kutokuwa na Amani<br>02 – Hajui mtu yoyote<br>03 – Hatakuwa karibu<br>04 – Nyinginezo (Taja): ___________________________<br>88 DK 99 NR
11.5	Kuponi ngapi za kujisajili zilizopewa/gawia kwa mshiriki?	|__|__| idadi ya kuponi mshiriki alizopewa
11.6	Tafadhali jaza nambari za codi za kuponi:	|__|__| |__|__| Kuponi ya 1<br>|__|__| |__|__| Kuponi ya 2<br>|__|__| |__|__| Kuponi ya 3<br>|__|__| |__|__| Kuponi ya 4
